# A Common Phenotype Polymorphism in Mammalian Brains Defined by Concomitant Production of Prolactin and Growth Hormone

**DOI:** 10.1371/journal.pone.0149410

**Published:** 2016-02-19

**Authors:** Nathalie Daude, Inyoul Lee, Taek-Kyun Kim, Christopher Janus, John Paul Glaves, Hristina Gapeshina, Jing Yang, Brian D. Sykes, George A. Carlson, Leroy E. Hood, David Westaway

**Affiliations:** 1 Center for Prions and Protein Folding Diseases, University of Alberta, Edmonton, AB, Canada; 2 Institute for Systems Biology, 401 Terry Ave North, Seattle, WA, 98109, United States of America; 3 Center for Translational Research in Neurodegenerative Disease, College of Medicine, University of Florida, Gainesville, FL, 32611, United States of America; 4 Department of Biochemistry, University of Alberta, Edmonton, AB, Canada; 5 Mclaughlin Research Institute, 1520 23rd Street South, Great Falls, MT, 59405, United States of America; University of Rouen, France, FRANCE

## Abstract

Pituitary Prolactin (PRL) and Growth Hormone (GH) are separately controlled and sub-serve different purposes. Surprisingly, we demonstrate that extra-pituitary expression in the adult mammalian central nervous system (CNS) is coordinated at mRNA and protein levels. However this was not a uniform effect within populations, such that wide inter-individual variation was superimposed on coordinate PRL/GH expression. Up to 44% of individuals in healthy cohorts of mice and rats showed protein levels above the norm and coordinated expression of PRL and GH transcripts above baseline occurred in the amygdala, frontal lobe and hippocampus of 10% of human subjects. High levels of PRL and GH present in *post mortem* tissue were often presaged by altered responses in fear conditioning and stress induced hyperthermia behavioral tests. Our data define a common phenotype polymorphism in healthy mammalian brains, and, given the pleiotropic effects known for circulating PRL and GH, further consequences of coordinated CNS over-expression may await discovery.

## Introduction

The growth hormone gene family includes growth hormone (GH) itself (also known as somatotrophin), prolactin (PRL) and the placental lactogens. Since sharing residual similarities in their amino acid sequences these proteins (for simplicity referred to as GH and PRL, respectively) are thought to have arisen from an ancestral gene by duplication [1]; divergence of the PRL and GH lineages from this gene is inferred to have taken place some 400 million years ago [2, 3]. GH and PRL are single-copy genes in rodents, whereas there are 4 further GH-like genes in human (reviewed in [4]).

GH and PRL are abundant products of the anterior pituitary, being produced by somatotroph and lactotroph cells respectively. Besides having endocrine effects on several target organs these polypeptide hormones have pleiotropic biological effects [5–7] and can enter the central nervous system (CNS) from the circulation via the choroid plexus. In addition, separate analyses have been performed for GH and for PRL that have been taken to indicate their synthesis by CNS neurons [8–12]. Importantly, disruption of the hypothalamic-pituitary axis by hypophysectomy or by ablation of pituitary cells by the chemical bromocriptine [13] has little effect on these CNS sites of expression. The conclusion of a mode of action independent of the pituitary is underscored by the ontogeny of expression of extrapituitary GH preceding that of development of the anterior pituitary and detectable circulating hormone [14, 15].

While linked secretion of GH and PRL in postnatal life has been denoted as an area of interest in the context of malignancy [16], during neurological studies of mutant mice [17–19], we were puzzled by two seeming “anomalies” present within our healthy control samples: first, coordinated expression of extrapituitary CNS transcripts of GH and PRL arising from these functionally diverged and non-syntenic genes, and second, marked animal to animal variation in the level of coordinate expression. These findings were confirmed in additional cohorts of adult wild type (wt) mice and rats maintained under standard housing conditions and without any pharmacological interventions. The findings were then further extended by defining protein expression above baseline in a subset (up to 44%) of animals of each species. These expression patterns had corollaries in altered behavior in paradigms involving stress response. As we could also demonstrate coordinate expression of GH and PRL within CNS tissue of control human subjects without neurologic diseases, this effect may be widely dispersed within the mammalian kingdom. Since “phenotype polymorphism” is a term used in the field of animal behavior and has been used for effects that may be of non-Mendelian origin [20, 21] we have used this terminology to refer to the changes in PRL/GH molecules.

## Material and Methods

### Animals

All animals used in this study were under standard laboratory conditions with a 12 hrs light/dark cycle with food and water *ad libitum*. Animal cohorts were housed at the University of Alberta (UA), the University of Lethbridge (UL), the McLaughlin Research Institute, Great Falls MT (MRI), and the University of Florida (UF), Gainesville FLA. Animals used were 6 to 49 week-old males and females. FVB/NCr at MRI, UF and UA, C57/129 at UA, BalbC/C57/129, CD1 and C57Bl/6J at UA, B6.I, Tg(Mo*Prnp*^a^)4053 at MRI, 129S2/SvPasCrL (129) at UF and rat Long-Evans at UL. B6.I are C57BL/6.I-1 and FVB-Tg(Mo*Prnp*^a^)4053 (Tg4053) mice described previously [22]. All the other mice and rats are wild type. Cross of FVB/NCr x 129 have been injected intracerebroventricularly with AAV2-eGFP or AAV2-BRI2-Stop, as described [23, 24]. The mice used in this part of our study were part of additional control cohorts set up for a different experiment, focussing on age-related changes in context- and cue-conditioned fear memories, and none of the data generated by these control cohorts have been published. We (CJ) recently demonstrated that neonatal cryoanesthesia does not affect physical development of mice and their cognitive function in the adulthood [25]. **Ethics statement:** Animal protocols were approved by the Institutional Animal Care and Use Committees at UA, UL, MRI and UF. Experiments at the UA and UL were in accordance with the Canadian Council on Animal Care. MRI and UF are fully accredited by AAALAC International experiments were carried out in accordance with the Guide for the Care and Use of Laboratory Animals (NIH, USPHS).

### Western blot analyses

Brains of adult mice were homogenized in 0.01M sodium bicarbonate buffer, augmented with protease inhibitors (Complete tablet, Roche Diagnostics). Whole extracts (50 μg of total protein) were subject to 14% SDS-PAGE gel electrophoresis and transferred to a polyvinylidene difluoride (PVDF) membrane (Millipore). After transfer, the membranes were blocked in 5% (w/v) non-fat milk in TBS-0.1% Tween-20 for 45 min at room temperature. The membranes were incubated overnight at 4°C probed first either with rabbit polyclonal antibody against mouse GH or PRL (dilution 1:500) (purchased from DR. A.F. Parlow, National Hormone and Peptide Program, Torrance, CA, USA), and then re-probed with anti beta-actin (Sigma). Secondary antibody used was goat anti-rabbit horseradish peroxidase-conjugated (Bio-Rad) diluted at a 1:10000 dilution. Immunodetection using the enhanced chemiluminescence (ECL) method (Pierce) was performed according to the manufacturer’s instructions. Immunoreactive protein species were quantified using ImageQuant software using local background correction (Molecular Dynamics, USA). Statistical analysis of the data was performed with a Fisher’s exact test with p-value <0.05 considered significant.

### Quantitative real-time RT-PCR

Trizol extraction of total RNA from 1 μg of tissue was performed according to the manufacturer's instructions. RNA integrity number (RIN) was measured by the Agilent® 2100 Bioanalyzer. Samples were treated with DNase I (New England Biolabs), and reverse transcribed with random hexamer primers. First-strand cDNA was used as the template for PCR amplification for real-time qPCR. Real-time qPCR was carried out in an iCycler iQ multicolor detection system (Bio-Rad; 1X 95°C for 5 min; 45X 95°C for 30 s, 57.2°C for 45 s, 72°C for 1 min; 1X 72°C for 5 min; 35X 65°C for 15 s). Primers used in the PCR are listed in **S1 Table**. Glyceraldehyde-3-phosphate dehydrogenase (GAPDH) was used as a control as it is not regulated in our microarray analysis. Reactions conditions were optimized to obtain amplification within the logarithmic phase of the reaction reproducibility. We also performed a melting curve analysis (temperature range 65–95°C) to check for the formation of primer-dimers and production of nonspecific products. Each reaction was performed in triplicate and threshold cycles (C_T_) were calculated using the second derivative of the reaction. The C_T_ of each gene was normalized against that of GAPDH. Fold changes were determined using the −ΔΔC_T_ method. Controls without RNA were performed to ensure that amplification of products was specific and was not due to non-specific contamination. Real-time qPCR has been performed according to the MIQE guidelines [26].

### Bioinformatics and microarray analysis

For the microarray analysis of mouse samples, we used a time-course gene expression data of mouse brains that we previously reported and described in systems biology analyses [17]. Briefly, from three to four biological replicates per experimental group, total RNAs were isolated with Trizol reagent further purified on RNeasy columns (Qiagen, USA). The quality of RNA samples was evaluated by using Bioanalyzer (Agilent, USA) and 5 μg of total RNA was reverse transcribed to generate double-stranded cDNAs. Corresponding biotin-labeled cRNAs were fragmented and hybridized to the GeneChip Mouse Genome 430 2.0 Array (Affymetrix, USA) containing 39,000 transcripts. After 16 hours of hybridization, the arrays were washed, stained with streptavidin, washed and scanned as per manufacturer's instructions. Array data were background-subtracted and normalized using gcRMA algorithm [27]. The quality of array data was assessed for overall correlation among biological replicates within each group and between groups; qualified array data was then deposited in prion disease database (PDDB; [18]). Pearson’s correlation coefficients and p-values between any of two probe sets in the PDDB were computed to determine the relationship of GH and PRL gene expression. Probe sets for microarrays are listed in **S2 Table**. Further mammalian microarray data were extracted from bioGPS http://biogps.org/#goto=welcome [28, 29] for analysis of rat samples and from http://www.ncbi.nlm.nih.gov/geoprofile for human samples (see **S4 Table** with all the latter human samples represent individuals with no overt sign of neurologic disease). For miRNAs we searched six databases: http://www.microrna.org/microrna/home.do, [30]; http://mirdb.org/miRDB/, [31, 32]; http://www.targetscan.org/; http://www.ebi.ac.uk/enright-srv/microcosm/htdocs/targets/v5/; http://www.umm.uni-heidelberg.de/apps/zmf/mirwalk/, [33]; http://mirnamap.mbc.nctu.edu.tw, [34]. For analysis of transcription factor (TF) binding sites, DNA sequences 5000 bp upstream of the initiation of transcription start were assessed for GH, PRL, POMC and CGA genes both in human and mouse using Alibaba2.1 (http://www.gene-regulation.com/pub/programs/alibaba2/index.html); this algorithm predicts TF binding sites by constructing matrices from the TRANSFAC® 4.0 public site.

### miRNA microarray analysis of mouse brain RNA samples

miRNA expression of mouse brain samples was profiled with a two-channel Exiqon miRCURY LNA array, 6^th^ generation (Exiqon, Woburn, MA) following the manufacturer’s instructions. The arrays were scanned using an Agilent scanner, and the images were processed using Agilent Feature Extraction program. Raw miRNA profiling data of the samples were combined and normalized using the quantile normalization approach. Since different platforms were used to measure the expression level of mRNA and miRNA, each of the data was auto-scaled for comparison of these data. The auto-scaling was computed using a following formula. ${\tilde{X}}_{ij}=\frac{X_{ij}-\underline{X_{i}}}{SD(X_{i})}$, where i and j indicate a mouse and a gene/a miRNA, respectively and SD stands for standard deviation.

### Immunohistochemistry

Each specimen was fixed by immersion in neutrally buffered 10% formalin, dehydrated and processed in paraffin wax. After de-paraffination and rehydratation, 6 μM thick sections were blocked for endogenous peroxidase in 3% H_2_O_2_ and washed in PBS with 0.05% Tween-20. When necessary antigen retrieval was performed in 0.01M citrate buffer (pH 6.0) and autoclaved for 2 min at 121°C. Slides were then incubated with biotinylated anti-mouse GH (Novus Biological) at 1:500 dilution overnight at 4°C. After washes, they were incubated with horseradish peroxidase-streptavidin and developed with 3,3’-diaminobenzidine tetrahydrochloride (DAB) (Dako, ARK™ kits). The slides were counterstained with Mayer’s hematoxylin and mounted. The slides were then analyzed using digital slide scanner Nanozoomer XR (Hamamatsu, SZK, Japan). For immunofluorescence, the same procedure was applied except that primary antibodies were non-biotinylated anti-mouse GH (Novus Biological) and PRL (Thermo Fisher), both at a dilution 1:500 and the secondary antibodies used were Alexa Fluor^®^ 488 Goat Anti-Mouse and Alexa Fluor^®^ 594 Goat Anti-Mouse (Invitrogen). Slides images were processed with the following uniform parameters: gamma correction 2.5; brightness 100%, and contrast 120% for all images developed with DAB and gamma correction 1.2; brightness 80%; contrast 120%; colour balance, red 200, green 145 and blue 100 for immunofluorescent analyses).

### Experimental design for behavioral tests

Experimental mice were handled once per fortnight after weaning and daily during a week preceding the first behavioral test. We used the hand-cupping method for handling acclimatization; the mice were scooped up by hand, allowed to stay on the experimenter’s cupped hand and were transferred to the holding cage or weighing container, without direct physical restraint [35]; physical restraint associated with picking the mice up by the tail increases anxiety, which may confound the results of subsequent cognitive tests [35]. The behavioral test battery for both age cohorts included an initial assessment of the physical conditions of mice using a SHIRPA screen (supplementary files), followed by spatial reference memory version of Morris water maze (MWM) test and delay fear conditioning (FC) test. Additionally, the older age cohort underwent a stress-induced hyperthermia (SIH) test after the completion of FC test. Following the behavioral evaluation, the mice were sacrificed and their brain were extracted, weighed and stored in -80°C for further analyses.

*Morris water maze (MWM) test*: Mice were trained in a water maze test, 140 cm in diameter, as described [36]. A *reference memory version* of the test was run for 5 consecutive days with 4, 60-sec, training trials per day. A mouse was released into water at semi-randomly chosen cardinal compass points (N, E, S, W [37]) and its swim path was recorded by image-tracking software (HVS Image). Dark, geometrical shapes (2–3 per wall), and two partitions separated experimenter, recording equipment, and a small cage rack from the testing area. The distance from the edge of the pool to the walls of the room or partitions was between 1 to 1.3 m. An escape platform, submerged 0.5 cm under water surface, was positioned in the center of the same NW quadrant of the pool (target quadrant, TQ) throughout the whole training phase. Memory bias for the platform location was evaluated in a probe trial (with escape platform removed) 24 hrs after the last day of training. The main variables analyzed during learning acquisition phase included search path (m), swim speed (m/sec), floating (swim below 0.05 m/sec), and thigmotaxis (wall-hugging swim) within 0.12 m distance from the wall of the pool. The spatial memory for the platform position was evaluated by the analysis of the search path in the TQ and by the annulus-crossing index (ACI). ACI represents number of crosses over the platform site in TQ adjusted for crosses over platform sites in the other three quadrants of the pool [37]. *Fear Conditioning test*: The test was performed as previously described [38]. A 4-chamber conditioning apparatus (Coulbourn Inst.) was located in a dedicated testing room. A tone (80 dB, pulse (6 clicks per sec), 30-sec duration) was used as conditioned stimulus (CS) and a 0.45 mA, 2sec foot shock, which co-terminated with a tone, as unconditioned stimulus (US). Mouse activity was recorded by FreezeFrame (Actimetrics) program, and freezing or immobility behavior, defined as cessation of all movements other than respiratory activity [39], indicative of fear memory of the association between CS and US, was analyzed off-line. Each mouse received 2 CS-US pairings separated by a 60-sec interval during one 5-min training session. After a day of recovery (D2), the contextual fear memory of mice was evaluated by exposing the mice to the context of the original training chamber (D3), and a day later (D4), the fear memory elicited by tone was evaluated in the modified context of the chamber (tone fear memory). In the tone test, the mice were allowed to explore the modified chamber for 3 min, followed by the 3-min tone presentation. Both context and tone tests were carried out in an extinction mode with no shock administered. *Stress-induced hyperthermia (SIH)*: The screen evaluates anticipatory anxiety reflected by increase in body temperature in response to the mild stress of handling with restraint [40]. Rectal temperature of a restraint by hand mouse is recorded at two intervals separated by 10 min. The positive difference (ΔT = T_2_–T_1_) reflects a hyperthermic response to the stress of restraint.

### Analysis of behavioral data

Since the control treatment (transfection with AAV2-eGFP or AAV2-BRI2-Stop) was not part of any *a priori* hypothesis-driven analyses related to the effect of hormones on behavior, we first evaluated whether the expression of eGFP and BRI2-Stop differentiate mice behavior. Since the groups did not differ significantly in any administered test, we combined the data across both treatments to increase the power of the analysis since two out of eight age x treatments cells had small number (n = 2) of mice of each sex. We used general linear model (GLM) of factorial ANOVA (Statistical Package for Social Sciences, SPSS v.21, Inc. Chicago), with hormones (GH and PRL), age (5 and 12 months) and sex as between subject, and learning and memory scores as within subject factors (whenever appropriate) to analyze the data. When necessary, degrees of freedom were adjusted by Greenhouse-Geisser epsilon correction for the heterogeneity of variance Bonferroni adjustment of α level (MODLSD Bonferroni t-tests, SPSS v21) was applied in multiple planned comparisons. Comparisons between two independent groups were done using Student *t*-test. The critical α level was set to 0.05, and all values in the text and figures represent means ± SEM.

## Results

### A GH/PRL phenotype polymorphism in mouse brain mRNAs

**Fig 1:**
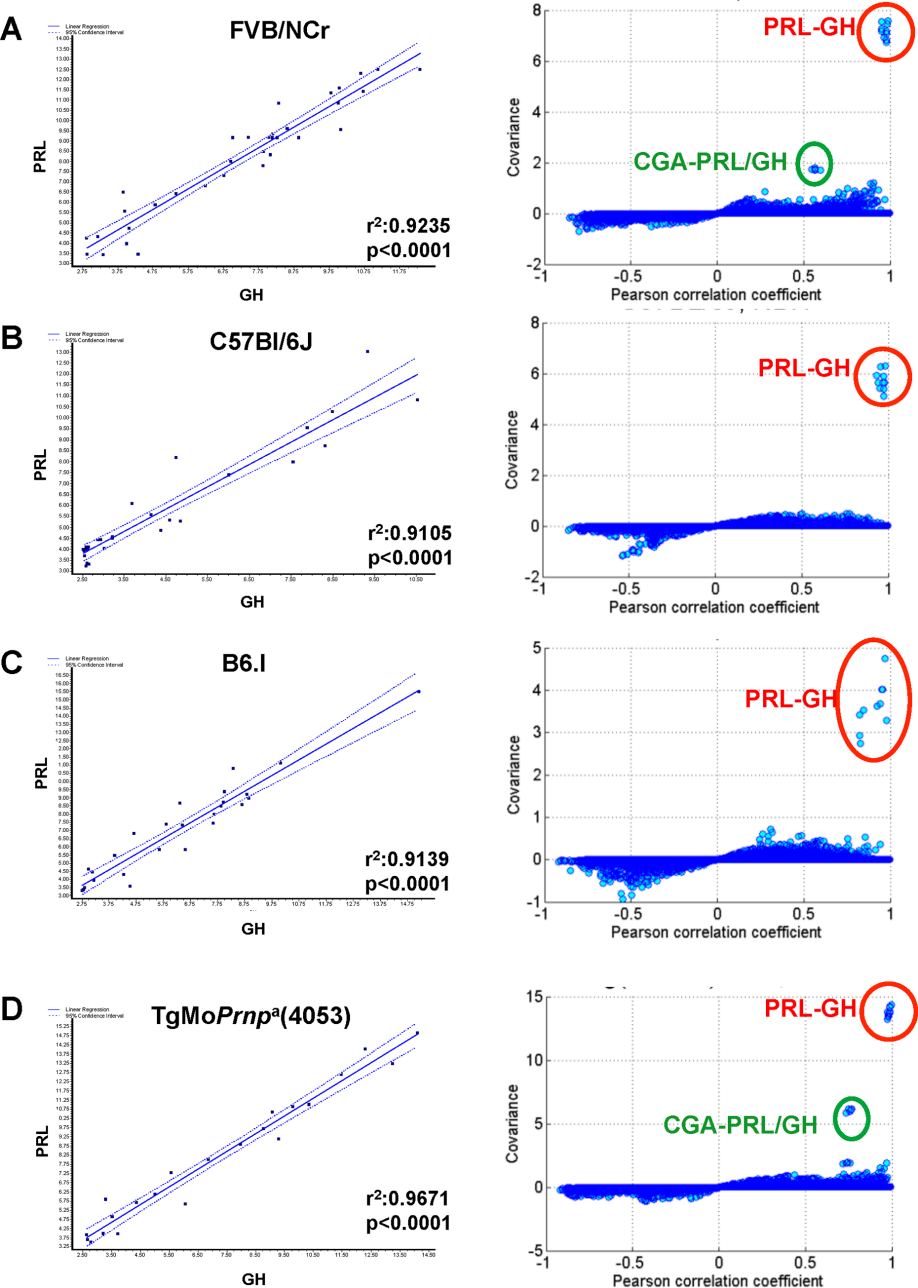
GH and PRL transcripts analyzed in healthy cohorts of four different mouse strains. Linear regression analyses of PRL (y-axis) and GH (x-axis) transcript levels in brain RNAs from (A) FVB/NCr, (B) C57Bl/6J, (C) B6.I, and (D) FVB-TgMo*Prnp*^a^(4053) mice are presented in the left-hand panels. Covariance (y-axis) versus correlation coefficient plots are shown in the right-hand panels; the co-regulated (outlier) behavior of PRL-GH and chorionic gonadotrophin alpha-chain (CGA) + PRL-GH is indicated within circled data points.

GH and PRL expression data for mRNA in brain analyzed by microarray is presented for 113 control animals from within the “prion database” [17, 18] (**Fig 1**). Specifically, these animals comprised negative controls for an infection experiment, being intracerebrally inoculated with a 30 microlitre volume of normal brain homogenate (because most of the injected volume is rapidly dispersed by bulk flow this procedure has no discernible impact apart from local scarring along the needle tract). These brain samples, as well as other rodent samples listed below, were all harvested during the light phase of a 12hrs light-dark cycle. RNA was purified from hemi-brains sectioned sagittally along the midline and therefore including all neuroanatomical areas present from the olfactory bulbs in an anterior position to the brain stem just prior to the commencement of the spinal cord. The RNA was copied to cDNA and biotinylated cRNAs were profiled using the Affymetrix 430 2.0 microarray platform. In these studies four probes represent GH and one probe represents PRL (**S2 Table**). Expression level of these probes were used to assess expression in wt FVB mice (**Fig 1A**, n = 34, range 49–189 days old (d)), C57Bl/6J mice (**Fig 1B**, n = 31, 49-196d), congenic mice carrying the b allele of *Prnp*, B6.I (**Fig 1C**, n = 26, 49-161d) and transgenic mice overexpressing the a allele of *Prnp*, TgMo*Prnp*^a^(4053) (**Fig 1D**, n = 22, 42-91d). In each case, and irrespective of the genotype we observed a remarkably correlated pattern of CNS expression of PRL and GH (all p-values <0.0001; r^2^ value range 0.91–0.97), even though these genes are unlinked and diverged >400 million years ago [41]. However, PRL and GH mRNAs measured in spleen tissue of the identical mice did not show the type of concordant expression pattern (**S1 Fig**) observed in the brain.

**Fig 2:**
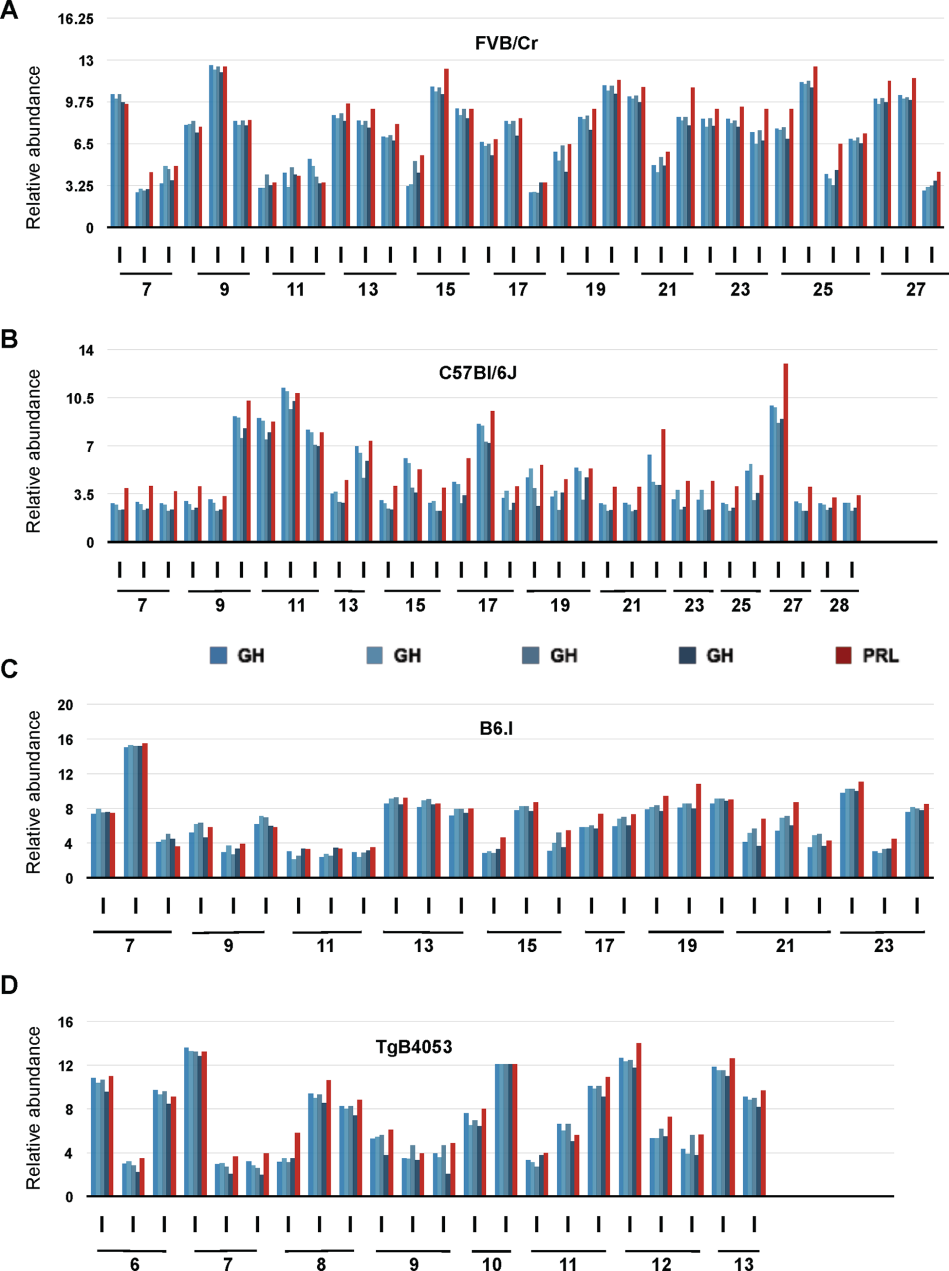
Microarray analysis of brain GH and PRL transcripts in 113 individual mice. Expression profiles of GH (shades of blue, 4 different probe sets) and PRL (red, 1 probe set) in brain of individual mice of different background during aging is shown. Dynamic gene expression profiles of (A) mice of FVB/NCr background aged from 7 to 27 weeks (n = 34), (B) mice of C57Bl/6J background, aged from 7 to 28 weeks (n = 31), (C) mice of B6.I *Prnp*^b^ background aged from 7 to 23 weeks (n = 26), (D) Data from Tg*Prnp*^a^(4053) mice are shown aged from 6 to 13 weeks (n = 22).

Beyond coordinated expression, a second phenomenon was also apparent in the data set, namely a marked individual-to-individual variation (**Fig 2**). Uniform trends in expression level as a function of the age were not apparent in any of the four groups and nor was the dynamic range of expression notably different from one strain to another. Insofar as this first dataset was derived from female mice (an experimental design to reduce fighting amongst animals housed for extended periods) it is possible that animal-to-animal variation in estrous was a salient variable. However, similar age-independent co-variation of GH and PRL was observed in other animal cohorts, including males, as described below.

To see whether this correlated expression effect was exclusive to PRL and GH we assessed the correlation between the expression levels of any given pair of hormone genes. Of 169 genes found in NCBI gene database using the search term “pituitary hormone” together with “*mus musculus*”, 134 genes (320 probes) were profiled in Affymetrix mouse 430 2.0 array (**S3 Table**). Any two probes were then randomly chosen and the correlation coefficient (CC) between them was computed. Some pairs of probes had high CC as a result of low, monotonous expression levels and to filter for this effect we generated scatter plots showing the relationship between covariance and CC (**Fig 1,** right-hand panels). The scatter plots show the best concordant expressional changes of PRL and GH in all four genetic backgrounds scrutinized. In addition, in the FVB/NCr background and a FVB/NCr-derived Tg line the CGA (the alpha chain of chorionic gonadotrophin was also correlated with PRL/GH expression **(Fig 1A and 1D; Tables 1** and **2)** and a significant effect also present for transcripts of POMC (pro-opiomelanocortin; a precursor protein that is processed to yield ACTH, beta-lipotropin, alpha- and gamma-melanocyte stimulating hormone and beta-endorphin) (**Tables 1** and **2**). Averaged across four GH probes versus PRL, p-values for three genetic backgrounds ranged from 2.15E-07 to 2.65E-27 and for PRL versus four GH probes the p-values ranged from 8.03E-07 to 5.35E-29 (**Tables 1** and **2**).

**Table 1:** Correlations in RNA expression levels referenced to averaged GH probes.

	Correlation coefficient			p-value		
	BL6	BL6.I	FVB	BL6	BL6.I	FVB
PRL	0.988631	0.986815	0.979375	2.03E-25	6.79E-19	8.50E-24
GH	0.990784	0.98084	0.989892	9.82E-27	4.03E-17	1.01E-28
GH	0.989866	0.975245	0.98984	3.87E-26	6.57E-16	1.10E-28
GH	0.993351	0.986407	0.992617	8.77E-29	9.47E-19	6.79E-31
GH	0.954185	0.844272	0.987593	9.76E-17	2.15E-07	2.65E-27
CGA	0.327339	0.50122	0.575843	0.07225	0.012593	3.66E-04
POMC1	0.442452	-0.07954	0.621936	0.012689	0.711778	8.62E-05
POMC1	0.628549	0.067002	0.622463	0.000153	0.755748	8.47E-05

**Table 2:** Correlations in RNA expression levels referenced to PRL probe.

	Correlation coefficient			p-value		
	BL6	BL6.I	FVB	BL6	BL6.I	FVB
PRL	1	1	1	0	0	0
GH	0.943286	0.821777	0.950777	2.01E-15	8.49E-07	7.67E-18
GH	0.932499	0.845896	0.99029	2.34E-14	1.93E-07	5.35E-29
GH	0.951668	0.82581	0.982907	2.09E-16	6.73E-07	4.32E-25
GH	0.954107	0.822749	0.979272	1.00E-16	8.03E-07	9.20E-24
CGA	0.333233	0.413031	0.563289	0.06697	0.044856	5.23E-04
POMC1	0.316795	0.075801	0.627506	0.082494	0.724814	7.12E-05
POMC1	0.471347	0.260798	0.626149	0.007437	0.21837	7.46E-05

Speaking further to the issue of specificity, we note that duplication events in the radiation of mammals have resulted in a number of PRL- and GH-like genes (in the latter case some being expressed in the placenta [42]). For the 23 gene duplication events for mouse GH and 9 gene duplication events for PRL, 11 of these gene paralogs are represented on Affymetrix mouse 430 gene chip arrays but in no instance did we find any correlation with GH and PRL transcript levels in the CNS.

### A GH/PRL mRNA phenotype polymorphism in rat brain sub-regions

**Fig 3:**
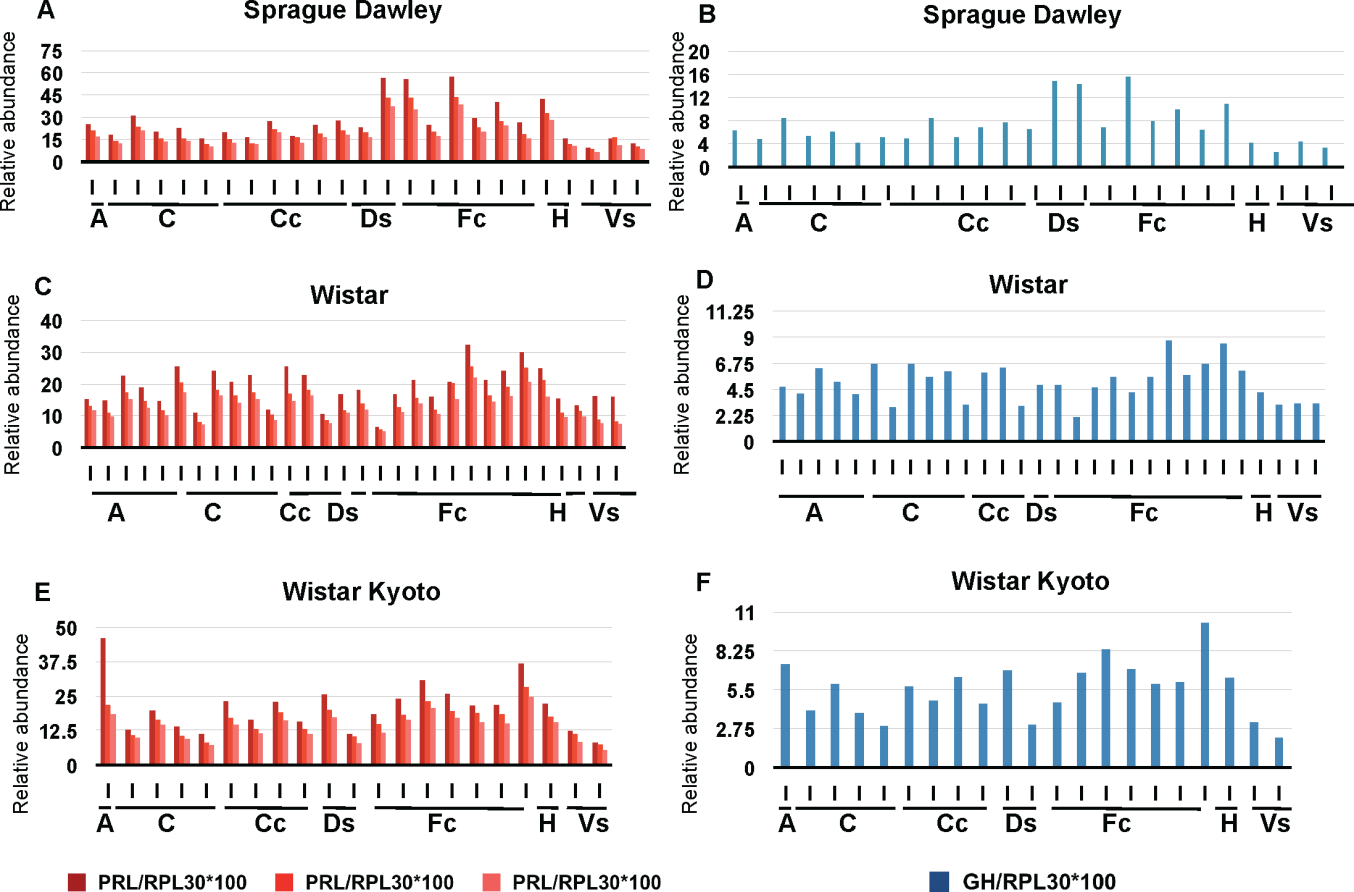
Inter-individual variation of GH and PRL transcripts in rat brain. Expression of PRL (A, C, E) and GH (B, D, F) in several brain regions (listed) in the strains Sprague Dawley (A, B), Wistar (C, D), and Wistar Kyoto (E, F). Three probes were used for PRL (shades of red), and one probe for GH (blue). Individual values were normalized against the housekeeping gene RPL30. A: Amygdala, C: cerebellum, Cc: Cerebral cortex, Ds: Dorsal striatum, Fc: Frontal cortex, H: Hippocampus, Vs: ventral striatum.

Following the results obtained in mice we considered whether concomitant expression of GH and PRL transcripts might apply to other species. To assess this possibility in the rat CNS, data were extracted from the bioGPS resource [28, 29] encompassing Sprague Dawley, Wistar, and Wistar Kyoto albino strains commonly used to model human physiology and diseases [29]. To exclude potential confounding effects of estrous, all animals used in this study were male (10 weeks old). Three oligonucleotide probes were used for PRL and one probe for GH (**Fig 3**, **S2 Table**). To facilitate comparisons between different analyses we searched for housekeeping genes with constant expression in rodent and human samples, with these candidates then checked further for invariance by use of RT-PCR: a total of five genes, RPL22, RPL27, RPS2,9 RPL30, and OAZ1 were defined by these means [43], with independent normalization to RPL30, OAZ1, and RPS29 being used for the following data. Results presented here were normalized to the housekeeping gene RPL30. The number of animals used in this study was 25 for the Sprague Dawley strain, 30 for the Wistar strain, and 21 for Wistar Kyoto strain. In this instance different neuroanatomical areas were dissected before RNA preparation: amygdala, cerebellum, cerebral cortex, dorsal striatum, frontal cortex, hippocampus, and ventral striatum. Relative abundance of PRL expression (**Fig 3A, 3C** and **3E**) was compared to the one of GH (**Fig 3B, 3D**, and **3F**), with statistical analysis shown in **S2 Fig** (all p-values are <0.001). Reminiscent of the data obtained in mice (**Fig 2**) we observed that signals for PRL and GH expression exhibited co-variation in all three strains and all six neuroanatomical regions examined. In the three strains studied, the relative expression of PRL was higher than GH; although this was not the case for the mouse dataset, this distinction might have arisen from the different platform and probes used in the two microarray analyses.

### A GH/PRL phenotype polymorphism for CNS protein expression in mice

**Fig 4:**
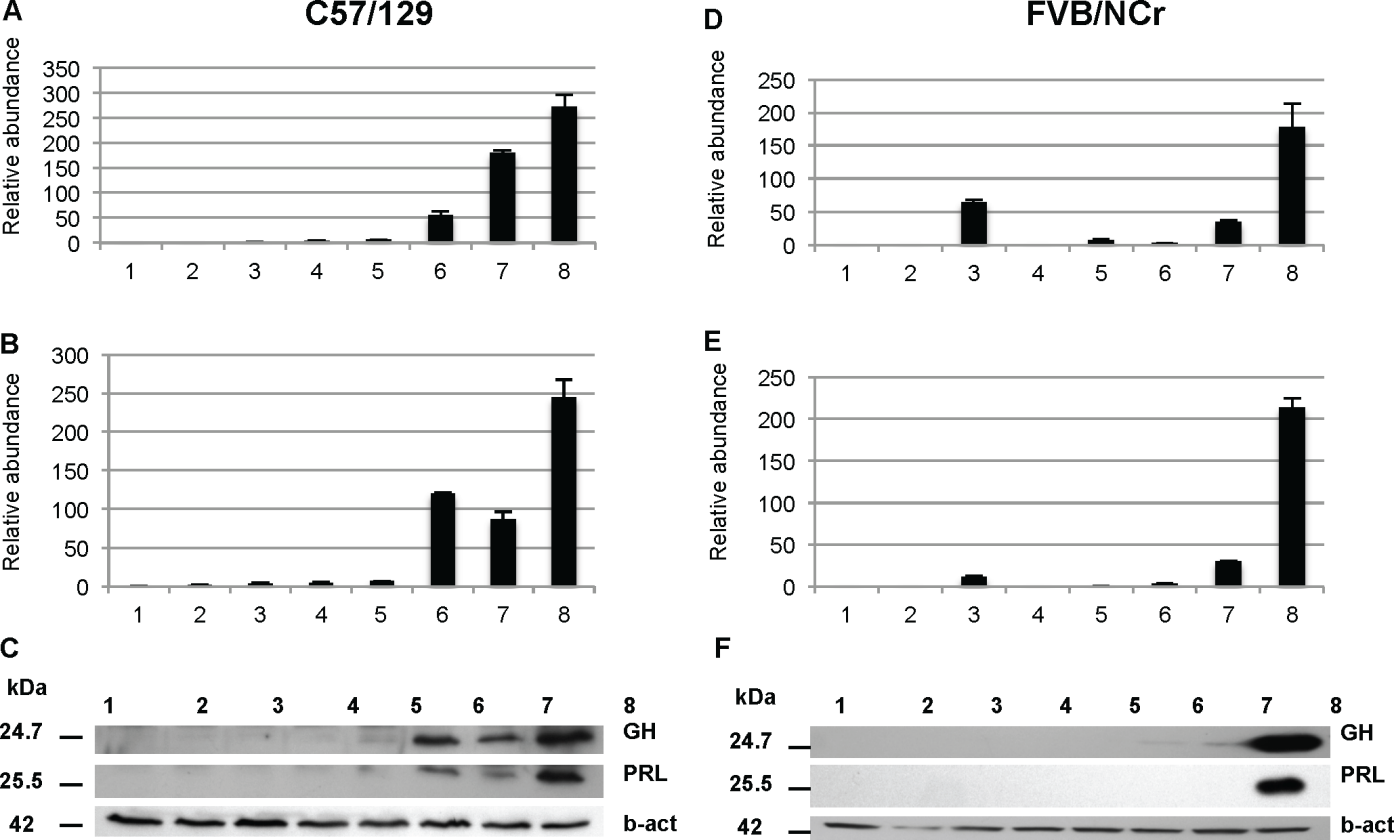
Parallel analyses of GH and PRL RNA and protein expression in mouse. Changes in mRNA expression, as measured by real-time qPCR, of GH (A, D) and PRL (B, E) in the brains of C57/129 (A,B) and FVB (D,E) wild type mice are presented. Numbers 1 to 8 correspond to individual animals. Real-time qPCR results are based on triplicate measurements (relative expression) for each sample. Immunoblots of the C57/129 (C) and FVB/NCr (F) strains were probed with anti GH, PRL, and beta-actin antibodies as described in Materials and Methods.

We next used parallel analyses from tissue of the same animals to ascertain whether variations in transcript levels produced commensurate changes in protein expression. To avoid potential confounding effects of estrous or chronological age, we examined male mice of the same age group (5 month-old). We used two different genetic backgrounds: C57Bl6/129 and FVB. Anaesthetized animals were first subjected to transcardiac perfusion with saline, then brains were removed, homogenized and used for the preparation of RNA or protein. In agreement with microarray analyses, individuals differed in their expression of GH mRNA (**Fig 4A and 4D**) as assessed by RT-PCR analyses following the MIQE guidelines [26] and with samples presented here by increasing order of expression of GH. Most animals displayed signals at the detection threshold of the techniques. However, on an individual-by-individual basis, for the animals where this was not so there was again a correspondence to expression of PRL transcripts, an effect that applied to both genetic backgrounds (C57/129, **Fig 4A** vs. **4B**; FVB, **Fig 4D** vs. **4E**). We then analyzed the protein level of GH and PRL in the same mice by immunoblotting with antibodies from the NIH National Hormone and Peptide Program. In mice from the C57/129 background (**Fig 4C**), the level of a ~24 kDa immunoreactive GH signal (predicted 24.7 kDa; UNIPROT) paralleled the pattern for mRNA abundance by RT-PCR. Concerning PRL, a ~26 kDa immunoreactive signal (predicted 25.5 kDa; UNIPROT) also paralleled mRNA expression. In the FVB strain (**Fig 4F**), we also observed that the highest levels expression of the protein coincided with the highest levels of mRNA (for both hormones). Taken together these results suggest that when the production of mRNA for both GH and PRL is elevated, these RNAs are translated into the corresponding protein products.

### Large-scale analyses of concomitant CNS expression of GH and PRL

**Fig 5:**
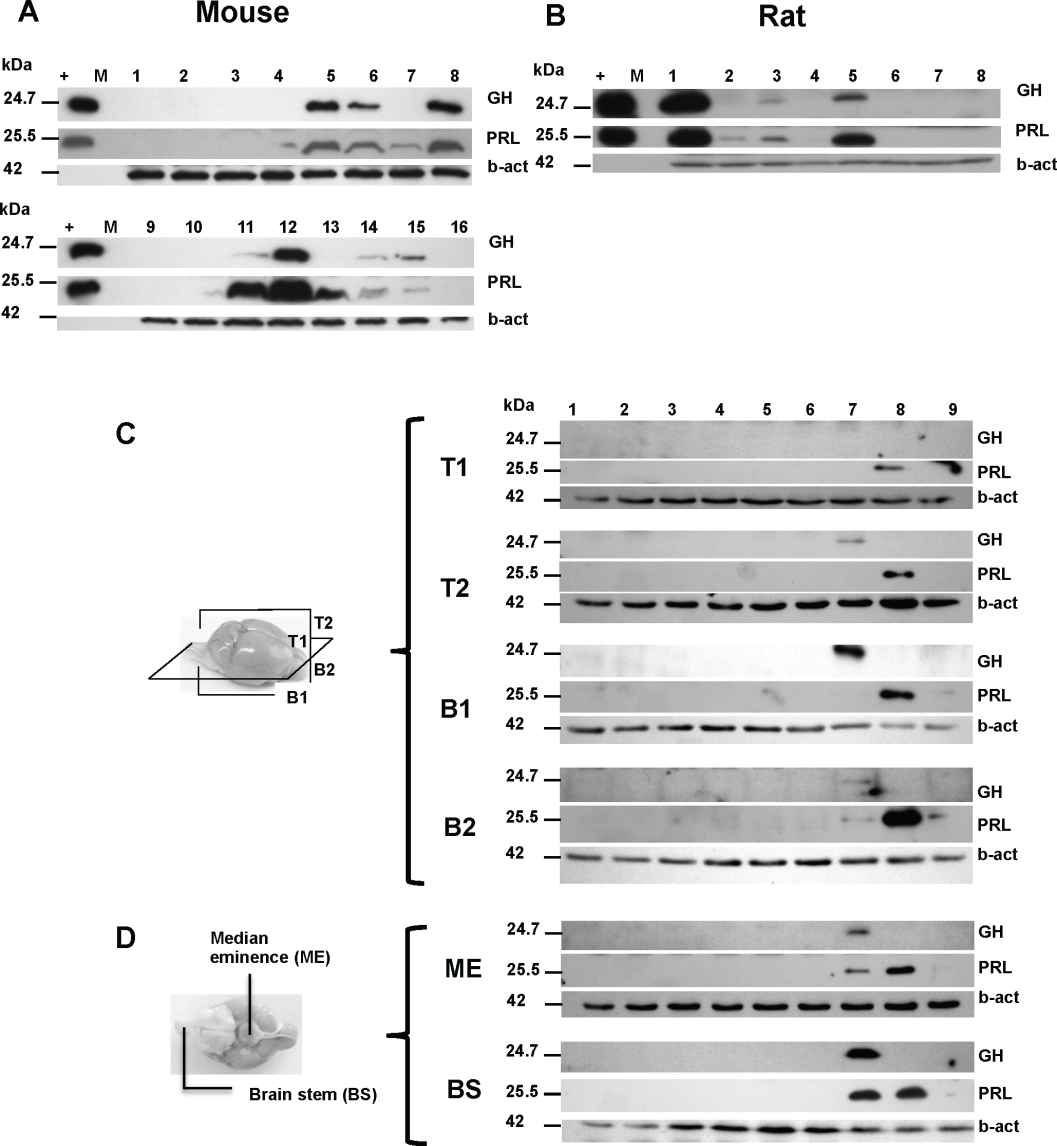
GH and PRL protein expression in a series of wt mouse and rat brains. Protein expression of GH and PRL in mouse (A) and rat (B) brains, respectively. 16 animals are represented for FVB/NCr mice and 8 for Long-Evans rat. The (+) lane represents 4 ng of pituitary extract as positive control. “M” represents molecular weight marker. Panels C and D represent the GH and PRL expression in dissected sub-region of mouse brain. (C) Mouse brains were cut horizontally and sagittally as depicted in the accompanying diagram to generate 6 types of samples: Upper left (T1), upper right (T2), lower left (B1), lower right (B2), median eminence (ME), and brain stem (BS). (D) Diagram represents the ventral aspect of the brain showing ME and BS. Expression of GH and PRL proteins are represented for each sub-region, as well as loading controls probed for beta-actin.

Having established a correspondence between transcript and protein levels we expanded our analyses of individual variation in GH and PRL protein expression in the brain to include 198 mice and 22 rats. A representative western blot of expression of GH and PRL in the brain of 16 mice and 8 rats is represented in **Fig 5A and 5B**. Within these larger sample sets we observed that the expression of GH and PRL showed gradations in expression from negative (detection limit of the chemiluminescent assay) to a variety of higher values. Bands were quantified using ImageQuant software and subdivided into 2 empirical categories: low- (less than 10% of the signal of the diluted pituitary extract used as an internal control in the experiment) or high- expressers. Using this principle there was a strong correlation between the high expression of GH versus high expression of PRL, with 0 to 44% of animals being high expressers, depending on the genetic background. Discordances with one hormone high but not the other, ranged from 7 to 19% (**Table 3**). There is no correlation between the CNS level of the hormones and their circulating levels in the same animals, as shown in **S6 Fig**. The number of rats analyzed by this method (n = 22) was smaller than for mice but the trend was the same; we found that 23% had high GH and 14% had high PRL, with discordant occurrences being 18%.

**Table 3:** Analysis of GH and PRL protein brains from 198 wild type mice and 22 rats.

Strain	Total #	Age range	GH		PRL		Co-expressor*	Discordant*
*Mouse*			Low*	High*	Low*	High*		
Balb/C/C57/129	15	234–283	87% (13)	13% (2)	93% (14)	7% (1)	93% (14)	7% (1)
CD1	29	47–162	100% (29)	0% (0)	90% (26)	10% (3)	90% (26)	10% (3)
FVB	154	66–345	57% (88)	43% (66)	56% (87)	44% (67)	81% (125)	19% (29)
*Rat*								
Long-Evans	22	171–219	77% (17)	23% (5)	86% (19)	14% (3)	82% (18)	18% (4)

• Decimal places were rounded of the nearest integer

• ¶ High GH and PRL or low GH and PRL

• (n) = number of animals

### Anatomical aspects of concomitant extrapituitary GH and PRL expression

We next performed analyses to confirm that the concomitant CNS expression of GH and PRL within brain lysates in a subset of animals does not reflect contamination by blood or by tissue areas with high levels of expression. As noted, animals were saline-perfused in order to dilute blood-borne hormone (or endogenous immunoglobulins which might react with anti-mouse secondary antibodies) from brain samples before homogenization into lysis buffer. Secondly, dissection of the brain into neuroanatomical regions was undertaken subsequent to removal of tissue from dorsal sectors (see diagrams, **Fig 5C and 5D**) and then subsequent to removal of the median eminence, which is in direct contact with the pituitary. The brain stem was also separated from the rest of the brain, as it is known to express high levels of peptides hormones [44, 45]. In this fashion the brains from nine FVB male mice were divided along a dorsal/ventral and horizontal midlines to produce sectors of tissue (“T1”, “T2”, “B1”, “B2”, **Fig 5C**; “ME”, “BS”, **Fig 5D*)*,** these sectors then being homogenized and analyzed for GH and PRL protein expression by western blot. In this study, samples (animals) 1–6 were found to be negative for both GH and PRL in every area examined. The seventh animal was positive in upper right, lower right and left quadrants, brain stem and median eminence for GH, whereas PRL expression was present in lower left, brain stem and median eminence. Sample 8 was negative for GH and positive for PRL in all areas examined. Sample 9 has PRL expression in the lower right and left sectors. In these analyses, although the median eminence is most closely juxtaposed to the pituitary, in 6/9 samples no signal was noted, arguing against systematic error of cross-contamination. Overall, these data demonstrate GH and PRL expression in one third of the animals at sites physically removed from the pituitary/median eminence/brain stem. Further, they reveal a degree of left-right symmetry in expression of the two hormones, either individually or in concert.

**Fig 6:**
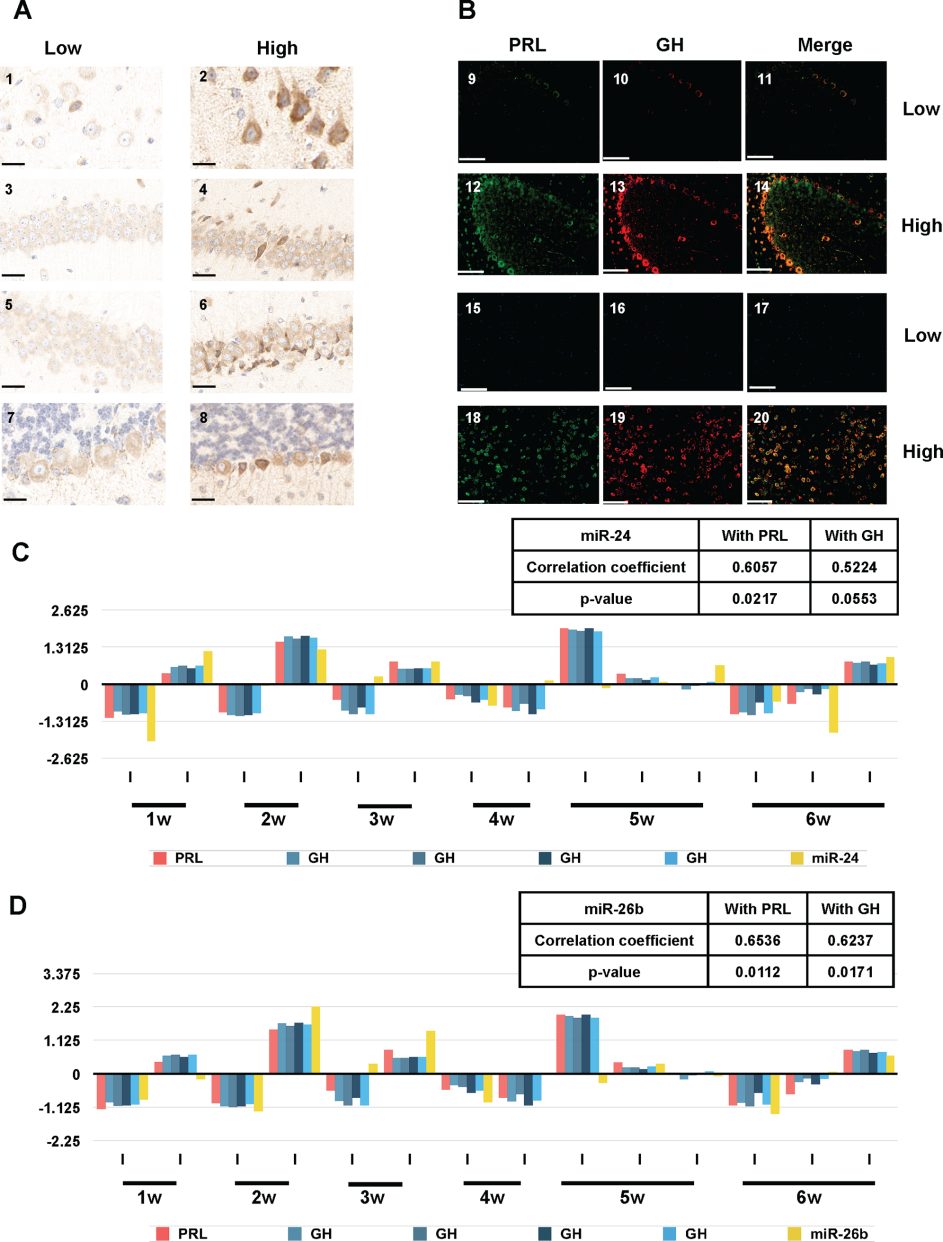
Immunohistochemical and miRNA analyses of mice with basal or elevated extra-pituitary GH/PRL expression. (A) Representative histological sections of brains wild type mice stained with anti-GH antibodies. Panels 1, 3, 5, and 7 correspond to the brain of an animal which is a low expresser of GH/PRL by western blot while panels 2, 4, 6, and 8 correspond to the brain of an animal which is defined as a high expresser of GH/PRL by western blot. Different regions of the brain are shown: cortex (1, 2), hippocampus CA1 (3, 4), hippocampus CA3 (5, 6), and cerebellum (7, 8). Scale bars represent 25 μM. (B) Fluorescent double staining for PRL (green, panels 9, 12, 15, 18), GH (red, panels 10, 13, 16, 19) and merged expression (orange, panels 11, 14, 17, 20) is presented. Panels 9–11 and 15–17 correspond to the brain of an animal which is low expresser of GH/PRL by western blot while panels 12–14 and 18–20 correspond to the brain of an animal which is defined as a high expresser of GH/PRL by western blot. Different regions of the brain are shown: cerebellum (9 to 14) and amygdala (15 to 20). Scale bars represent 50 μM. (C) Correlation between brain GH/PRL transcripts and miR-24 in a cohort of healthy FVB TgMo*Prnp*^a^ (4053) mice. (D) Correlation between brain GH/PRL transcripts and miR-26b in a cohort of healthy FVB TgMo*Prnp*^a^ (4053) mice. The y-axes in panels C and D represent relative abundance (as per Figs 2 and 3) of the miRNAs.

Building on the observation of bilateral expression in some animals, mice in a new cohort were anaesthetized, perfused and sacrificed, with brains then removed and cut sagittally. One half was prepared for western blot analysis and second half was fixed in formalin, paraffin-embedded and cut in a semi-coronally. Samples, which were either positive or negative for GH expression by western blot, were then analyzed in immunohistochemistry using monoclonal antibodies against GH (**Fig 6A**). Panels **1, 3, 5** and **7** represent brain sections from animals negative for the expression of GH by western blot, whereas panels **2, 4, 6**, and **8** represent positives. The most striking differences between low-and high-expresser mice were in the cerebral cortex (**1, 2**), hippocampus CA1 and CA3 neurons (**3, 4** and **5, 6**) and cerebellar Purkinje cells (**7, 8**). Often, cells having a greater degree of immunostaining were located adjacent to cells with background levels of staining (**Fig 6A**, panels **4**, **6** and **8**). Co-labeling experiments with two monoclonal antibodies were used to assess whether GH-producing cells also express PRL. In **Fig 6B**, panels **9** to **14** represent a view of cerebellum and panels **15** to **20** represent a view of the amygdala. We noted that cells with a strong expression of GH (cerebellar Purkinje cells, panel **13**) and amygdala (panel **19**) often show strong expression of PRL (panels **12** and **18**), and this is presented in overlay (panels **14** and **20**). Conversely, cells showing low levels of GH (panels **10, 16**) show an equally low level of PRL staining (panels **9, 15**).

### Transcription factors and extra-pituitary GH and PRL expression

With changes in PRL/GH gene expression applying to transcripts as well as proteins (**Figs 1–5**), a posttranslational effect can be excluded. Beyond PRL/GH, co-expression of CGA (normally expressed in pituitary thyrotroph and gonadotroph lineages) or POMC (normally expressed in corticotroph/melanotroph lineages) was also observed (**Tables 1 and 2**). As these genes lie on different chromosomes (**S5 Table**), it becomes unlikely that the phenotype polymorphism derives from co-inheritance of (hypothetical) allelic polymorphisms in *cis* regulatory elements for each of these genes. Instead, we investigated the possibility that mammals can relax mechanisms that dampen extra-pituitary CNS expression of these genes via changes in the co-regulatory networks that govern miRNAs and chromatin accessibility/transcription factors [46]. We addressed this possibility by converging *in silico* interrogation of (a) curated TF interactome data and six miRNA interactome databases (microRNA.orf, miRTarBase, miRDB, miRNAMap, TargetScan and miRBase used in conjunction with metacore and IPA software) (**S6 Table**) with (b) population-wide *in vivo* data using brain cRNAs from a cohort of mice assessed in miRNA arrays and RNA microarrays that included the TF genes of interest.

The standard deviation of all the miRNA profiles was examined to identify miRNAs with varied intensity values reflecting variations among individual mice. We selected the top 9 miRNAs (miR-200a, miR-200b, miR-182, miR-429, miR-183, miR-200c, miR-141, miR-96 and miR-24) showing the highest standard deviations. Of the 9 miRNAs, miR-24 shows the best correlation with GH and PRL (**Fig 6C**). Correlation analysis shows the miR-26b expression is also related to GH and PRL transcript levels (**Fig 6D**), with relationships between this microRNA and TF’s discussed below.

### Behavioral correlates of the CNS phenotype polymorphism

*Ante mortem* metabolomic profiling of urine of low- and high-expresser mice revealed a number of differences (**S8 Fig**), suggesting the high expression phenomenon can have a phenotypic impact. With regards to CNS-related outcome measures, since GH and PRL are considered pleiotropic with effects upon many endpoint measures [7, 47] we reasoned that behavioral analyses might offer a broader phenotypic view of CNS function to detect *in vivo* differences caused by the additional hormones present in some animals (and also noting that phenotype polymorphisms are apparent in studies of animal behavior [20]). To this end we performed *post hoc* protein expression determinations on coded brain samples from a cohort of mice that had been phenotyped previously and comprised negative controls for other studies [23, 48]. From a cohort of 54 animals we defined 30 animals with baseline expression (“low”) and 19 animals as having concomitant high expression (“high”) of GH and PRL proteins. A further 5 animals had discordant expression of CNS GH and PRL (in agreement with data presented in **Table 1**) but, due to the small size of this sample group, were not considered further.

### Spatial reference memory in the MWM test

**Fig 7:**
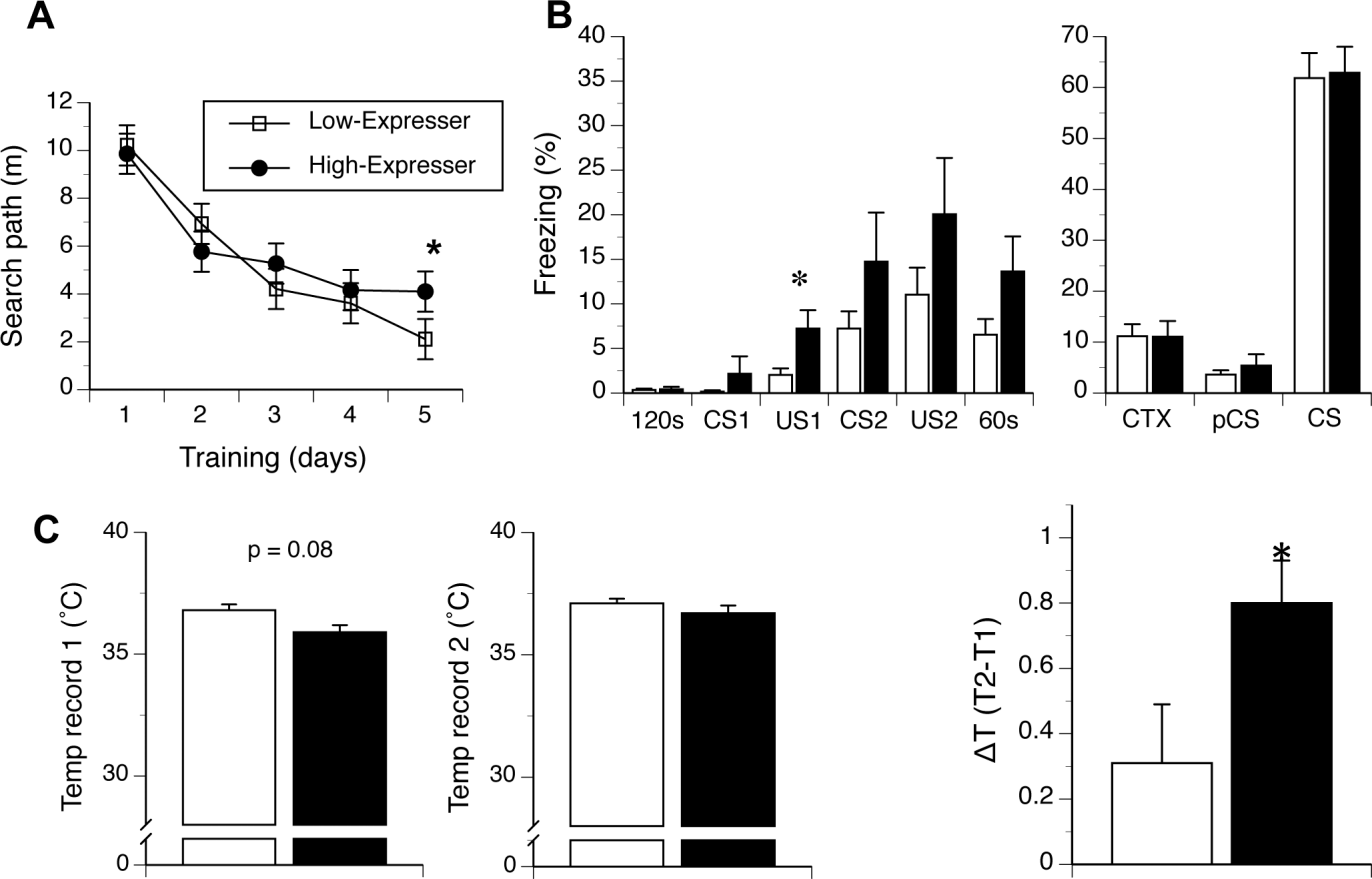
Behavioral analysis of mice with basal or elevated extra-pituitary GH/PRL expression. Differential expression of PRL and GH differentiates the behavior of 129FVB F1 mice. (A) High GH/PRL expressing mice showed a tendency of longer swim paths during leaning acquisition phase of the water maze test. (B) Left panel. High-expresser mice (black shading) showed longer freezing response to the first foot shock and following conditioned stimulus and unconditioned stimulus pairings (“CS-US”) during training in fear conditioning test. Right panel. The expression of PRL and GH did not differentiated conditioned context (CTX) or tone fear memory of the mice. (C) Left panel. High-expresser mice (black shading) showed a tendency of lower basal core body temperature in the stress-induced hyperthermia (SIH) test. Middle panel. Core body temperature during the second recording (T2) was comparable between the groups. Right Panel. High-expresser mice showed increased hyperthermia (ΔT) in response to the restrain stress. 120 sec– 2 min period of training chamber exploration; US–unconditioned stimulus; CS conditioned stimulus; 60 sec– 1 min recovery time.

Search paths for the location of hidden platform during learning acquisition phase of the MWM test did not reveal significant differences between hormone expression level (i.e. low versus high), age or gender, or in 2- and 3-way interactions involving these factors. All mice shortened the search path to reach the escape hidden platform during training (F(4,136) = 41.2, p < 0.001, **Fig 7A**). However, the decrease in the search path was affected by hormone expression (F(4,136) = 3.0, p < 0.05, hormones by path interaction). *Post-hoc* analysis revealed that the high expressing mice showed longer search paths in the second half of training, which reached significance at α = 0.05 on day 5 (t(21) = -2.5, p < 0.05, **Fig 7A,** degrees of freedom adjusted for the inequality of variances). No other interaction effects were significant (see also **S4 Fig**).

### Fear Conditioning (FC) test

Performance of mice in the FC test is shown in **Fig 7B**. Low- and high-expressers did not differ in their initial exploration of the training chamber, and none of the other factors or interactions between them was significant at the α = 0.05. Also, no differences were found between hormone expression, age and gender in the response to the presentation of the first tone (conditioned stimulus 1, CS1), indicating that the tone used in the experiment presented a neutral stimulus. Next, we compared response to a foot shock exposure and the consequent phases of training, to characterize immediate associations between cage context/tone and the application of a foot shock. Multivariate analysis (MANOVA) revealed significant effects of phase of training (F(3,102) = 8.6, p < 0.001), phase by sex and phase by hormone class by age interactions (F(3, 102) = 4.6, p < 0.05; F(3,102) = 3.3, p < 0.05, respectively). While no between-subject factors were significant, the hormone expression by age by sex interaction bordered significance (F(1,34) = 3.0, p = 0.09). *Post-hoc* analysis revealed that high-expresser mice showed a longer freezing reaction following the first exposure to a foot shock. (F(1,34) = 4.2, p < 0.05) and a trend towards longer freezing following the second exposure to a foot shock. High-expresser mice also showed a trend towards longer freezing during the second presentation of a tone (conditioned stimulus 2, CS2) and during the final 60s recovery period (**Fig 7B,** left panel). Hormone expression levels did not affect the context test (“CTX”) nor either component of the tone test (pre-conditioned stimulus, pCS, and CS tone test (**Fig 7B,** right panel). Also, age and sex factors did affect the development of conditioned memories, and none of the interactions between the factors was significant at α = 0.05. As an internal control for the validity of this assay the difference in freezing between the phase preceding the tone (pCS) and tone test (CS) (F(1,34) = 326.1), was significant, p < 0.001 (**Fig 7B,** right panel), indicating specificity of the conditioned freezing response to the tone stimulus.

### Stress-induced hyperthermia (SIH) test

Basal (“T1”), re-sampled (“T2”) core body temperatures as well as the change in temperature due to restraint (ΔT) of high- and low-hormone-expressing mice are shown in **Fig 7C**. Analysis of basal temperature and its change after 10 min of restraint revealed a gender effect (F(1,22) = 6.9, p < 0.02) and a significant difference between the recording times (F(1,22) = 20.0, p < 0.001). The change between T1 and T2 time points also depended on the hormone expression level (F(1,22) = 4.6, p < 0.05, temperature records by hormone expression interaction effect). High-expresser mice showed a tendency of lower basal body temperature than the low-hormones-expressing mice (35.9 ± 0.3°C *versus* 36.8 ± 0.2°C, F(1,22) = 3.3, p = 0.08, **Fig 7C**); however, they also showed higher increase in the body temperature after the second restraint than low-expresser mice (36.7 ± 0.03°C and 37.1 ± 0.2°C for high and low expressing groups, respectively, **Fig 7C**). Consequently, high-expresser mice showed a significantly higher hyperthermic effect in response to immobilization by physical restraint (F(1,22) = 4.6, p < 0.05 with no other factors or interactions were significant at α = 0.05).

### A GH/PRL phenotype polymorphism present within Human brains

A last set of analyses of human brains was designed to assess the broader applicability of the molecular polymorphism. Brain areas from 22 females (23–92 years) and 25 males (25–81 years) from subjects without neurological or other disease were analyzed for expression of PRL and GH mRNAs (http://www.ncbi.nlm.nih.gov/geoprofile); these areas corresponded to accumbens nucleus, amygdala, caudate nucleus, cerebellum, cerebral cortex, dorsolateral prefrontal cortex, frontal lobe, hippocampus, hypothalamus, lateral substantia nigra, medial substantia nigra, medulla, midbrain, occipital lobe, orbitofrontal cortex, parietal lobe, prefrontal cortex, putamen, substantia nigra, subthalamic nucleus, superior frontal gyrus, temporal lobe, thalamus, trigeminal ganglia, ventral tegmental area and vestibular nuclei superior. These human samples are represented by case number: females from 1 to 22 and then males from 23 to 77; in both cases the samples are presented in increasing age order (see **S4 Table**).

**Fig 8:**
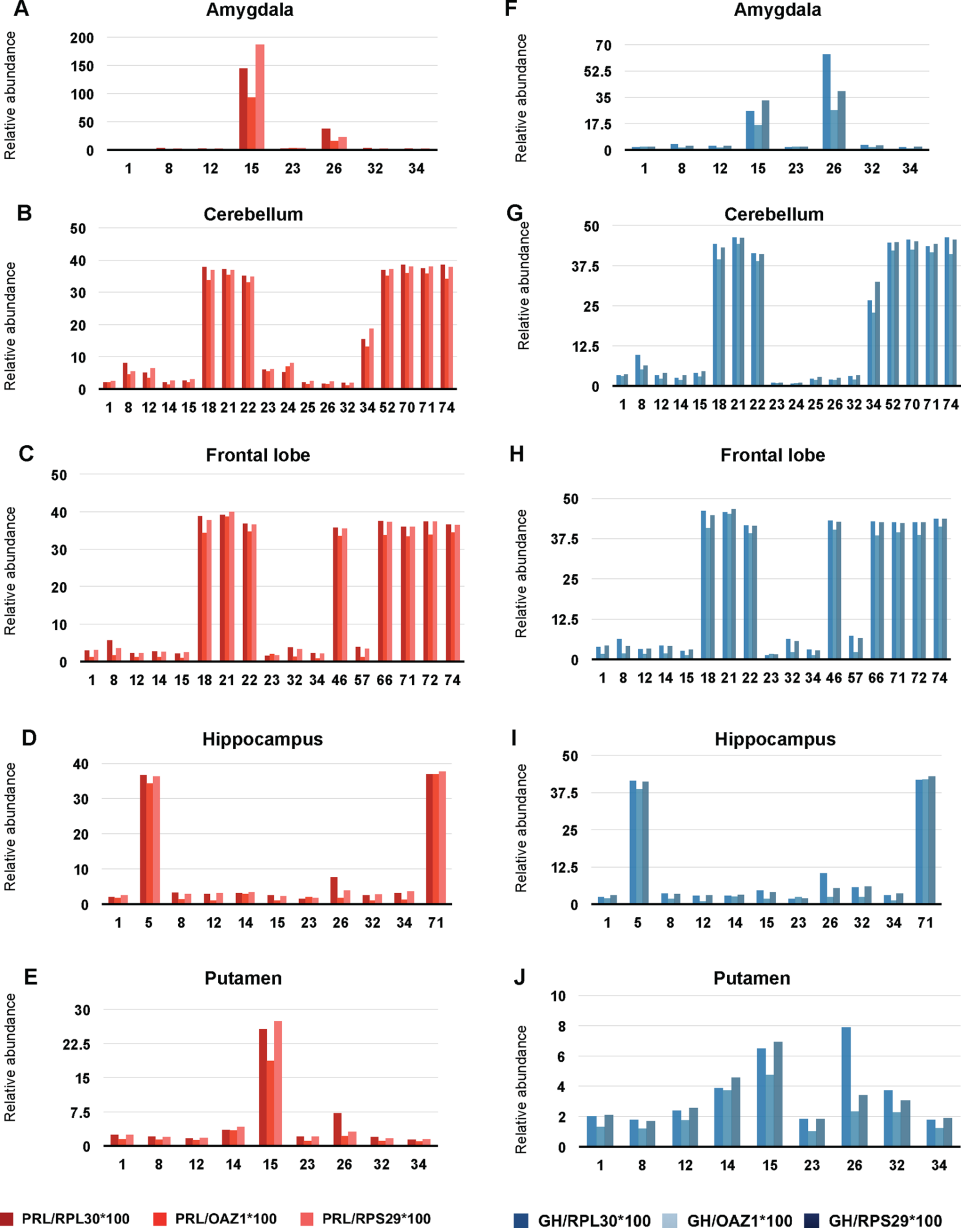
Microarray analysis of human brain regions reveals elevated extra-pituitary GH/PRL expression in a subset of subjects. PRL and GH expression in different brain regions of healthy humans. Both PRL (A, B, C, D, E, different shades of red) and GH (F, G, H, I, J, different shades of blue) have been normalized to 3 different housekeeping genes (RPL30, OAZ1, and RPS29). Numbers on the abscissa represent individual cases. 1 to 22 are female and 23 to 77 are male. Samples are shown in increasing order of age. Different regions of the brain are shown: amygdala (A, F), cerebellum (B, G), frontal lobe (C, H), hippocampus (D, I) and putamen (E, J).

While the levels of both GH and PRL transcripts were near the assay baseline and/or lacking significant variation for 34 subjects within twenty neuroanatomical regions, the remaining 13 subjects (28%) presented with stronger signals, these being present in amygdala (**Fig 8A and 8F**), cerebellum (**Fig 8B and 8G**), frontal lobe (**Fig 8C and 8H**), hippocampus (**Fig 8D and 8I**), and putamen (**Fig 8E and 8J**). Within these subjects, concomitant expression of GH and PRL was the norm (e.g., amygdala, **Fig 8A and 8F**) and concomitant increased expression could be present within different neuroanatomical regions of the same subject (e.g., amygdala and putamen for subject 15; amygdala, hippocampus and putamen for subject 26). Statistical analyses for these coordinated changes in gene expression define a p-value of 0.1692 for amygdala, possibly due to the smaller number of samples analysed, but cerebellum, frontal lobe and hippocampus have p-values <0.001 and putamen has a p-value of 0.0096, all considered highly significant (**S3 Fig**).

## Discussion

### Evolutionary aspects of concomitant extra-pituitary GH/PRL expression

Our data speak to a conserved phenomenon of concomitant extrapituitary expression of trophic factors, factors that are more typically considered as products of the pituitary. This effect occurs in the brains of a subset of mice, rats and humans, and with technical aspects of our analyses and discussions of alternative explanations presented at length in S1 **Appendix I**. This phenotype polymorphism of the adult CNS is common, being present in 7–44% of animals in selected cohorts (**Table 3**); it is often present in young animals and is not associated with any type of CNS neoplasia visible by light microscopy (not shown). Notably, and in counter-distinction to experiments looking at the effects of sex hormones and castration upon GH mRNA produced in the hippocampus and hypothalamus [11, 45], we found the correlated expression of extrapituitary GH and PRL to be present in both genders and with the corresponding proteins being detectable in a subset of animals by standard western blotting procedures (**Figs 4 and 5**). While GH expression in the hippocampus can be increased by stresses such as electric shock [11] the effect reported here was apparent in rodents maintained under basal housing conditions. Others have inferred from the appearance of GH in neural tissue prior to pituitary differentiation that CNS tissue represents the ancestral site of hormone production [49] and our data can be aligned with this concept. Overall, we infer that the phenotype polymorphism is at minimum benign and, likely being of ancient provenance, might confer a selective advantage upon populations that harbour it.

### Routes to concomitant extra-pituitary GH/PRL expression

How might the coordinate overexpression effect arise? In terms of accepted aspects of the biology of pituitary GH there is a decline of GH/Insulin-like growth factor I axis with age [50], which, amongst other effects, induces a decline in neurogenesis; however we do not see a decrease with age in the expression of both hormones in high expresser rodents and a trend to the contrary was present in the human microarray data (**Fig 8**). Extra-pituitary expression of GH or PRL (as individually regulated entities) has been equated by some with a mechanism to redress deficits in signaling deriving from circulating hormones when pituitary function is attenuated [13, 14, 51–54] but since our experiments did not involve chemical or surgical ablation of pituitary function these concepts may be of limited utility. For the extra-pituitary expression of GH and PRL defined here in a subset of housed rodents or in human microarray data the more general question arises as to whether we are sampling outliers in a spectrum of physiological variation of gene expression. While we cannot exclude this is the case for the individual hormones, since GH and PRL are non-syntenic in all the mammalian species considered here and have diverged with few overlapping functions, concomitant GH + PRL overexpression present in our data (with discordances no greater than 19%; **Table 3**) argues for a coordinated biological phenomenon rather than coinciding outliers. In terms of cell-to-cell communication, the existence of co-expressing cells (**Fig 6**) argues against a paracrine effect within clusters of cells expressing one or other of the two hormones. Studies to take these immunohistochemical findings further will benefit from analyses of control tissues from animals genetically deficient in GH and PRL, as well as antibodies against defined GH and PRL epitopes used in conjunction with blocking peptide controls.

In terms of non-Mendelian mechanisms that might effect transcript abundance, imprinting represents a mechanism mediated by DNA methylation and histone acetylation that results in differential parental contributions to inheritance [55]. But, while imprinting of insulin-like growth factor II receptor gene represents a well-known example of this effect [56], the mouse chromosomal regions encoding the GH, PRL, CGA and POMC are not imprinted (**S5 Table**; (www.har.mrc.ac.uk/research/genomic_imprinting). Parallel changes in mRNA and protein (**Fig 4**) speak against a posttranslational effect in protein processing trafficking or secretion but, rather, support an effect upon transcript levels. While a distal promoter in the human PRL gene directs extrapituitary expression in mammary gland and adipose tissue [4, 57, 58], this DNA element is not present in rodents and hence unlikely to contribute to the concomitant expression phenomenon. We next considered TFs that can bind *cis*-regulatory elements in the proximal promoters sequences of GH, PRL and other anterior pituitary hormone genes, but for TF mRNAs assessed in our mouse microarray dataset we were unable to detect significant correlations with PRL and GH that there were consistent across all four genetic backgrounds. A following consideration concerned micro RNAs, short non-coding regulatory sequences that have become of increasing interest since the early 2000’s. Within the genetic background scanned for miRNA expression on Exiqon arrays, miR-24 and miR-26b were significantly correlated with GH and PRL, with miR-26b being reported to have a potential impact upon expression of the TF Pit-1 in GH3 cells by inhibiting the Pit-1 inhibitor called Lef-1 [59]. These miRNAs are promising candidates for future investigations but deeper mechanistic insight to define how the GH and PRL genes can become mis-regulated will likely need to draw upon transgenic resources.

In terms of the signalling pathways that might engaged in the presence of extrapituitary GH and PRL, the sites of expression that re-occur in our studies of three mammalian species include amygdala, cerebellum, frontal cortex and hippocampus (**Figs 3, 6** and **8**). When we analyzed mRNA expression of GH and PRL receptors (**S9 Fig**) we did not observe a corresponding co-variation, e.g. an up-regulation in high-expresser animals, leading to a consideration of documented aspects of basal receptor expression. For GH receptor, mRNA has been described in the dentate gyrus and the caudate putamen [60] and GH binding sites are reported in the putamen, hippocampus and cortex (as well as anticipated sites in the choroid plexus [61]). For PRL receptor, the sites known to be activated by blood-borne hormone include the periventricular hypothalamus [62, 63], but receptor expression has been described elsewhere, including the olfactory bulb and the retina [64, 65]. How hormone levels in animals with concomitant extrapituitary expression of GH/PRL align with established dose-response relationships and how they might act to transduce signals to drive associated downstream behavioral (**Fig 7**) and metabolomic (**S8 Fig**) changes remains to be documented.

### Functional consequences of concomitant Extra-pituitary GH/PRL expression

The foregoing comments notwithstanding, earlier research has attached importance to the study GH or PRL in the brain as they might relate individually to the process of neurogenesis, or events following brain injury and consequent behavioural changes, including anxiety responses [66–69]. Reasoning that behavioral tests might be sensitive indicators of altered CNS function, we went on to define three alterations in higher PRL and GH expressing mice. A longer immediate freezing response to foot-shock could reflect an enhanced anti-predatory response, which did not however affect memory of the association between the context or tone and the foot-shock (a similar facilitated acquisition of avoidance behavior was observed when circulating PRL levels were increased by tissue grafts [70]). Two other effects relate to stress. Beyond the acute stress of inescapable restraint, increased swim speed in the water maze might indicate heightened anxiety in high GH/PRL expressing mice; this type of effect is observed when wt animals are not habituated to handling before testing [35] or in tasks that are non-biological for mice (mice in contrast to rats are not natural swimmers) [71]. In the prior behavioral literature, stress is reported as an inducer of local GH synthesis in the CNS [11], while GH expression in aging is purported to have a negative effect insofar as aged GHR knock-out mice avoid a cognitive decline that becomes manifest in wt animals [72]. It should be mentioned that our experimental paradigm (repeated, sequential testing), could blunt the response of mice to the testing environment in each subsequent test, resulting in a non-significant effect of age [73]. In future studies the inclusion of naïve cohorts of mice tested following a cross-sectional design may provide additional insights into the relationships between GH and PRL expression in the CNS in response to experimental stress.

Lastly, because circulating PRL and GH are well known as being pleiotropic, with literally hundreds of documented outputs [7, 47], it is possible that behavioral assays in other domains will offer differentiation of the high expresser animals. Prolactin can drive neurogenesis [66, 69, 74] and given the role of prolactin in nurturing behaviors [75] and patterns of sexual activity [76], assays in these domains will be of interest. Moving beyond the paradigm of studying PRL functions in pregnancy, the behavior of PRL- or GH/PRL- overexpressing male mice may be a worthy point of departure for a new series of studies.

## Supporting Information

S1 ARRIVE Checklist Link to ARRIVE checklist. (PDF)

S1 Fig GH and PRL transcripts in spleen analyzed in healthy cohorts of four different mouse strains. (TIFF)

S2 Fig Linear regression between GH and PRL expression in rat brain. (TIFF)

S3 Fig Linear regression between GH and PRL expression in diverse human brain structures. (TIFF)

S4 Fig Other aspects of water maze performance for high-expressing GH/PRL mice. (TIFF)

S5 Fig Control western blot for antibody specificity. (TIFF)

S6 Fig Comparison of GH and PRL content in mouse brain versus serum. (TIFF)

S7 Fig Immunohistochemical analyses of mice with basal or elevated extra-pituitary GH/PRL expression. (TIFF)

S8 Fig Metabolomic analyses of mice with basal or elevated extra-pituitary GH/PRL expression. (TIFF)

S9 Fig Microarray analysis of brain GH and PRL receptors transcripts in 113 individual mice. (TIFF)

S1 Table Primer sequences used in qRT-PCR. (PDF)

S2 Table Microarray probe-sets. (PDF)

S3 Table 134 genes associated with GH and PRL. (PDF)

S4 Table Data on human controls. (PDF)

S5 Table Gene locations. (XLSX)

S6 Table miRNAs associated with expression of GH and PRL transcripts. (PDF)

S1 Appendix Extended discussion of alternative explanations for concomitant extrapituitary GH and PRL expression. (DOCX)
