# Supplementary figures and images for: A Common Phenotype Polymorphism in Mammalian Brains Defined by Concomitant Production of Prolactin and Growth Hormone
Source: PLoS One. 2016 Feb 19;11(2):e0149410. doi: 10.1371/journal.pone.0149410 (PMC4760942; doi:10.1371/journal.pone.0149410)

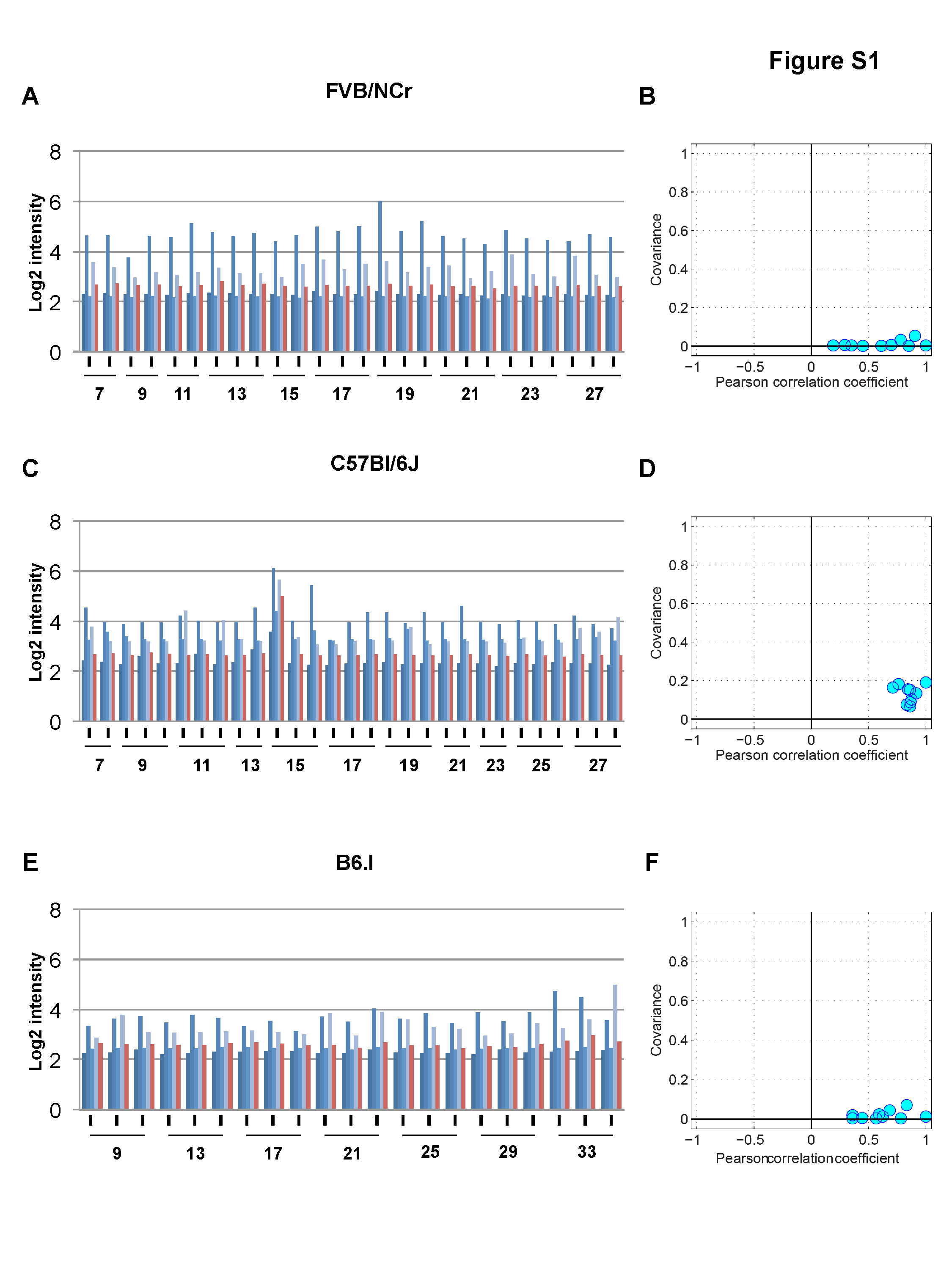

Supplement: S1 Fig — (TIFF) [file pone.0149410.s002.tiff]

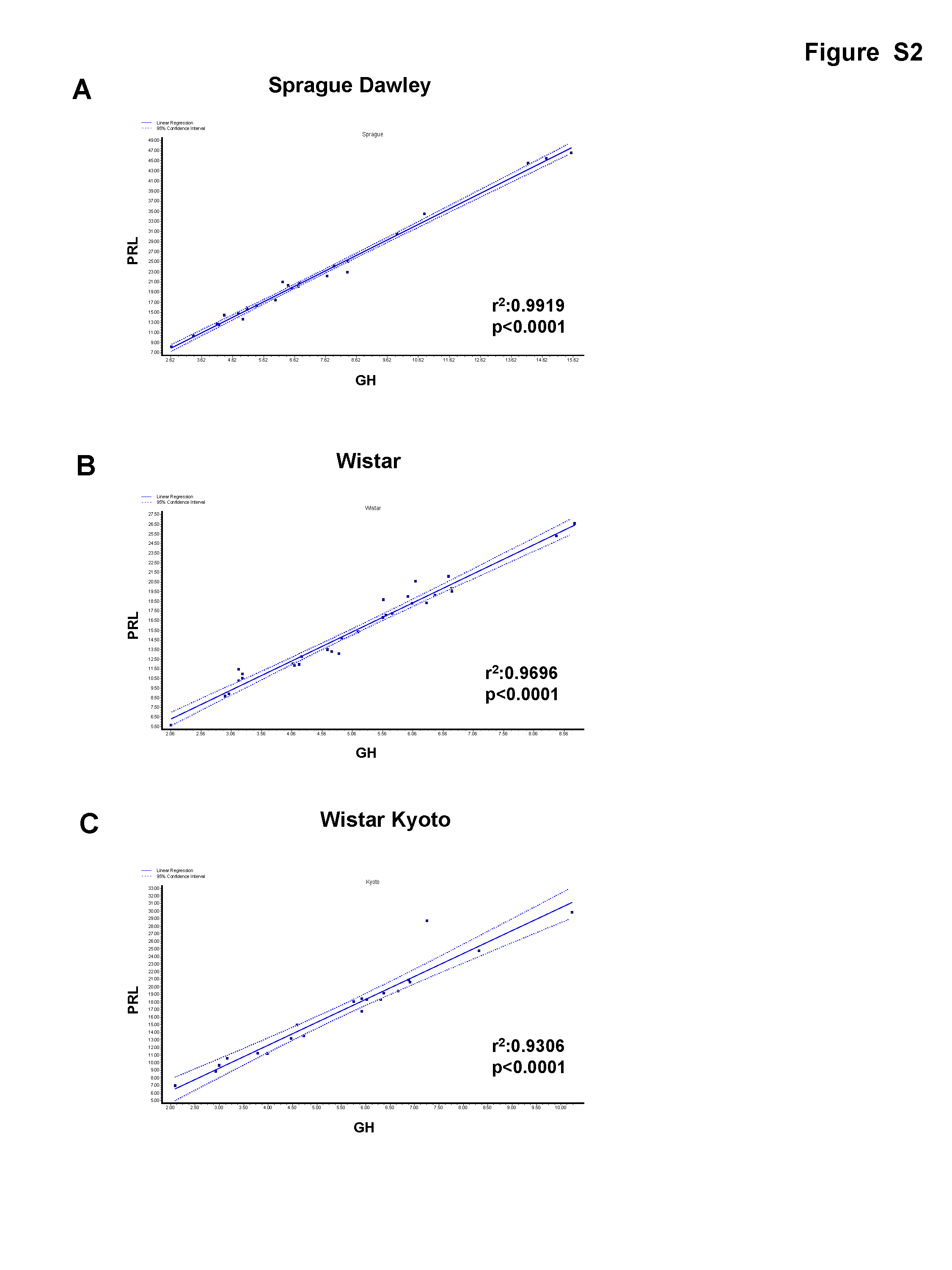

Supplement: S2 Fig — (TIFF) [file pone.0149410.s003.tiff]

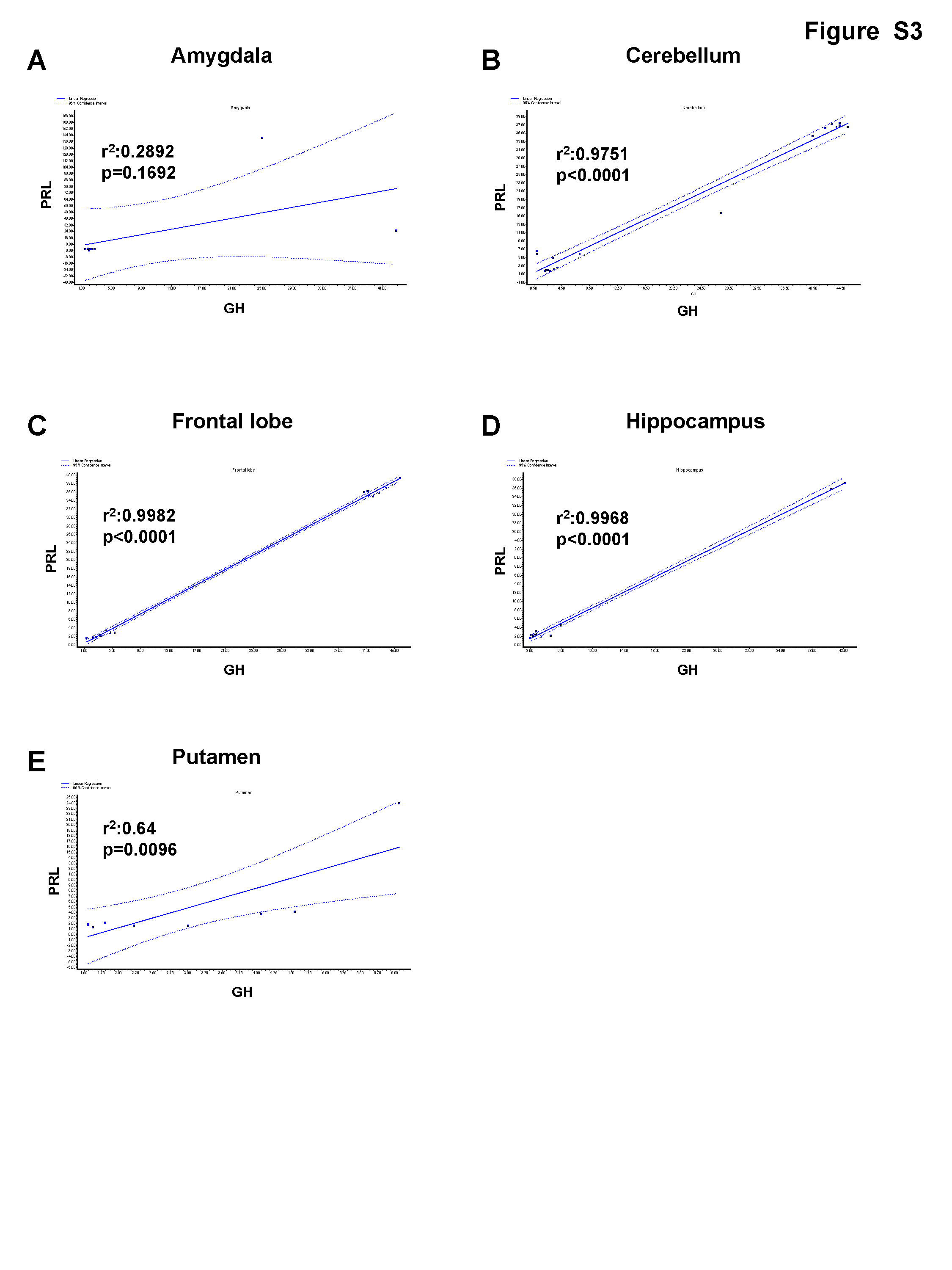

Supplement: S3 Fig — (TIFF) [file pone.0149410.s004.tiff]

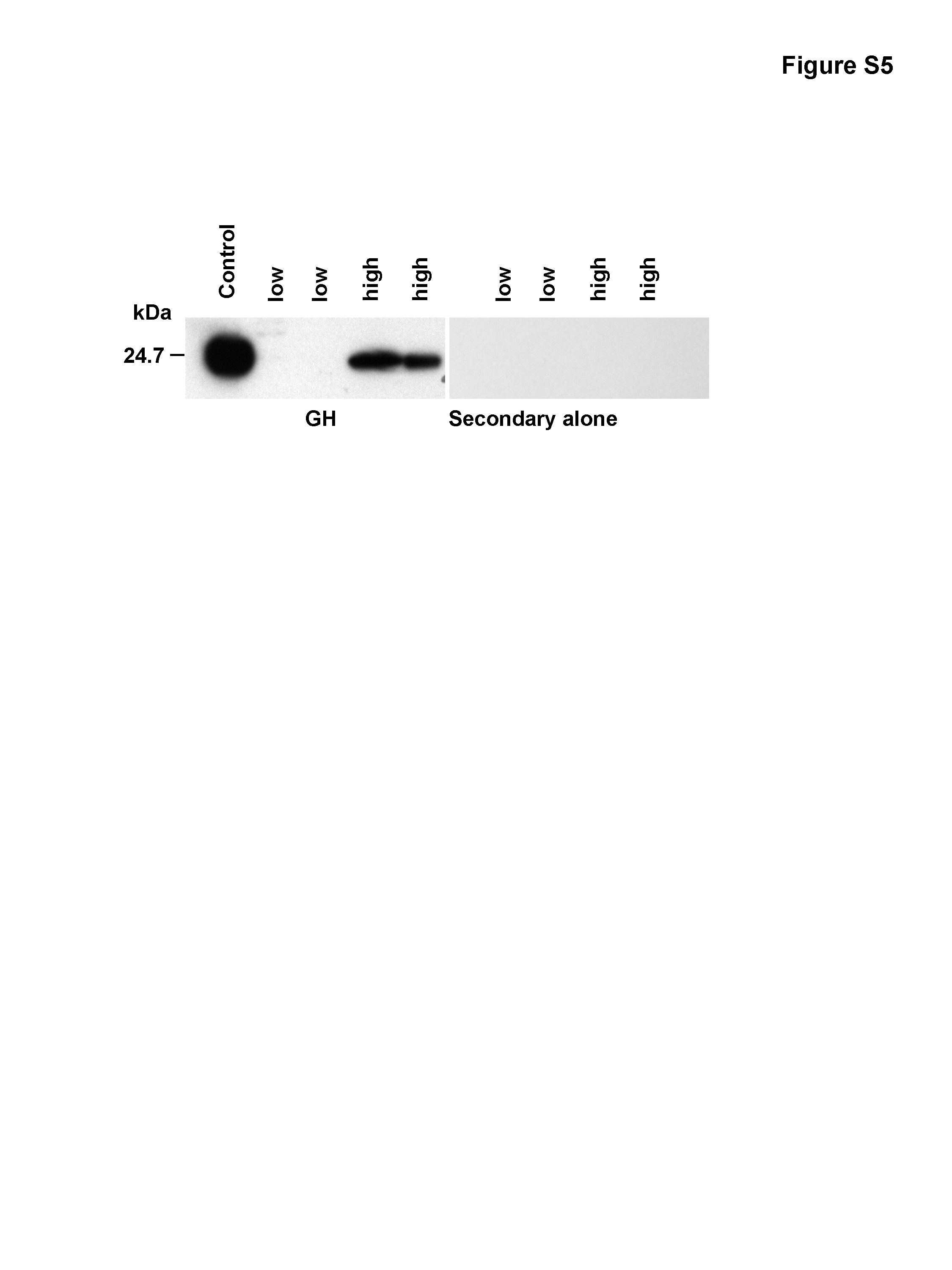

Supplement: S5 Fig — (TIFF) [file pone.0149410.s006.tiff]

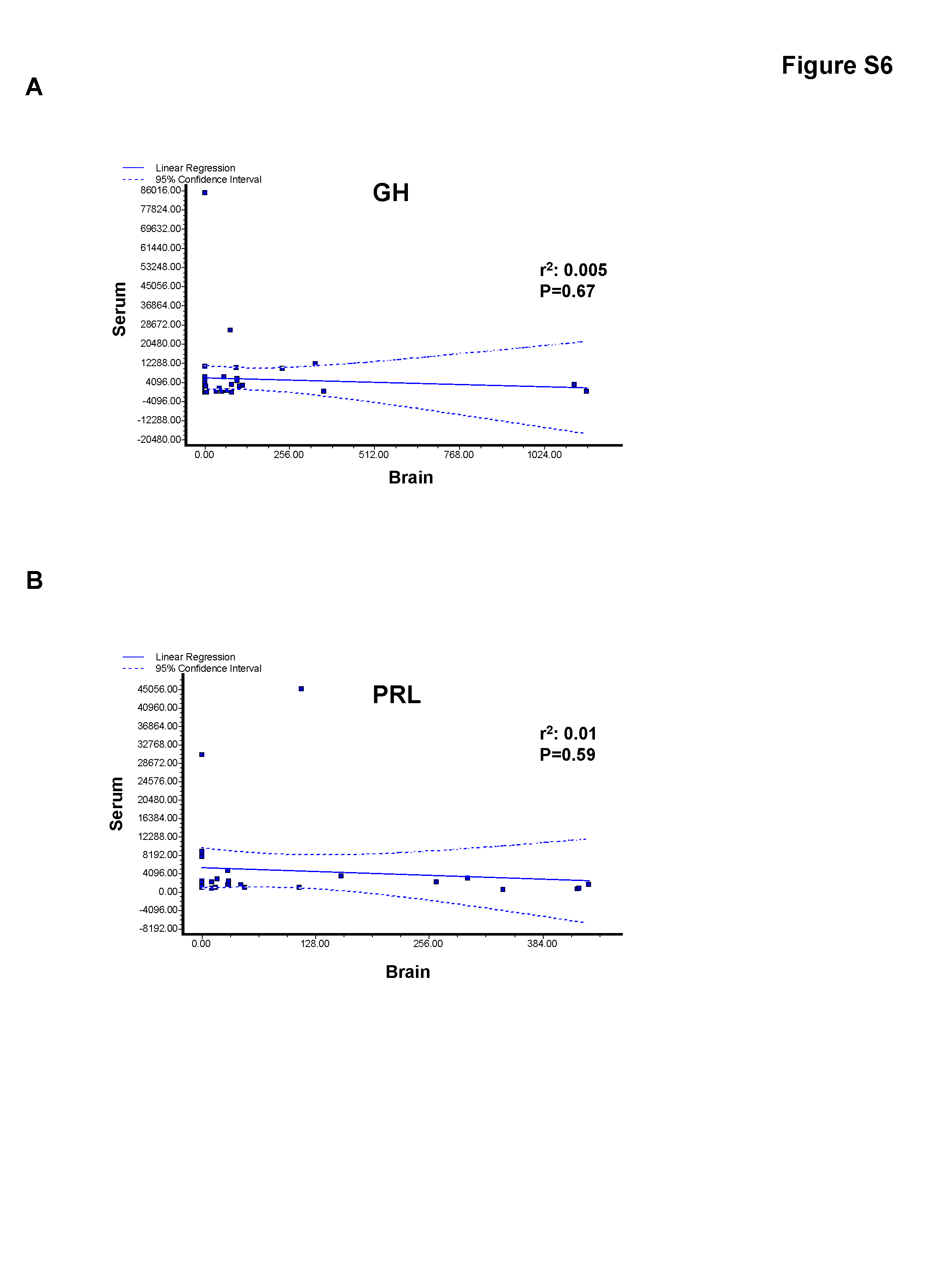

Supplement: S6 Fig — (TIFF) [file pone.0149410.s007.tiff]

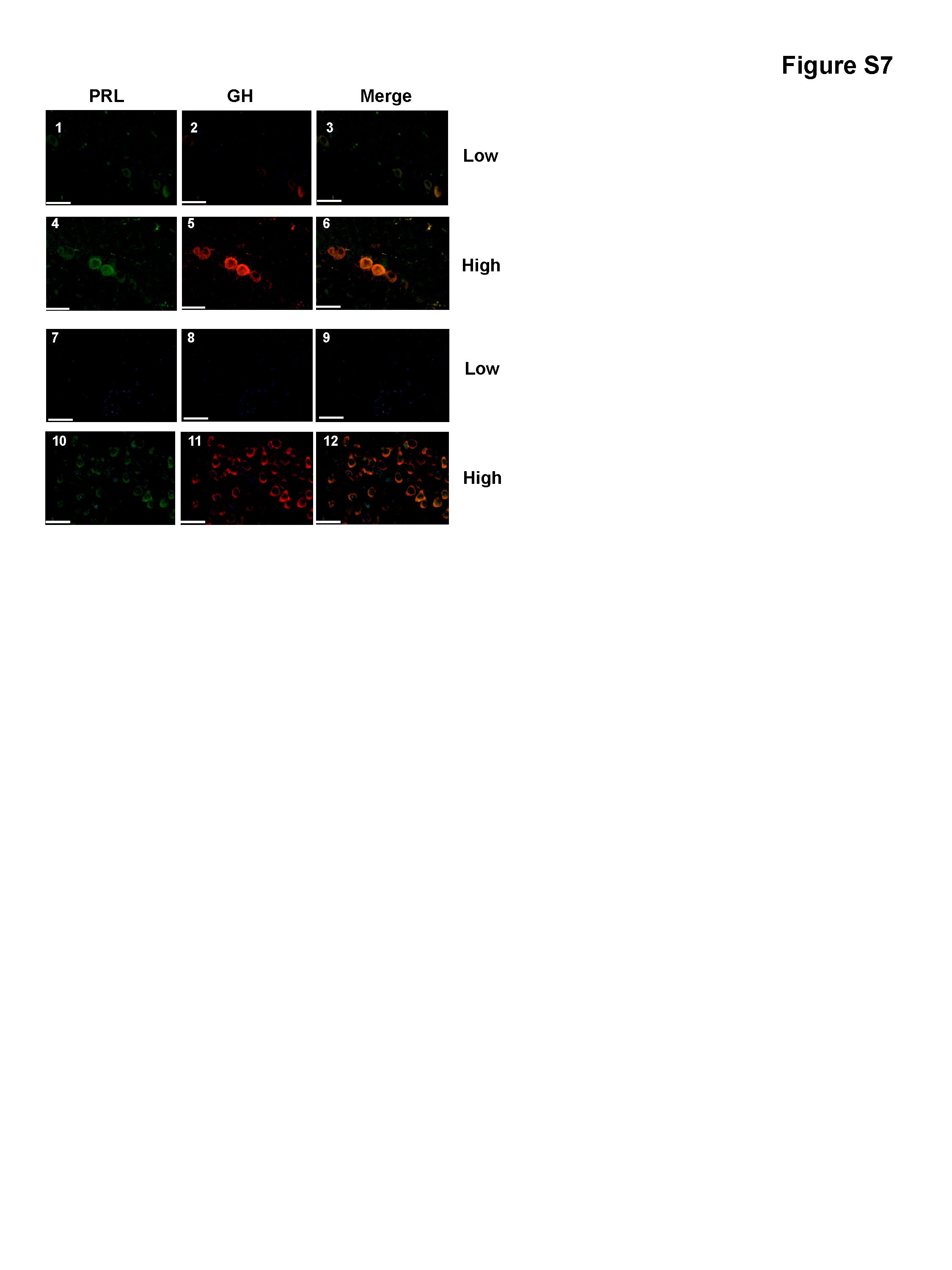

Supplement: S7 Fig — (TIFF) [file pone.0149410.s008.tiff]

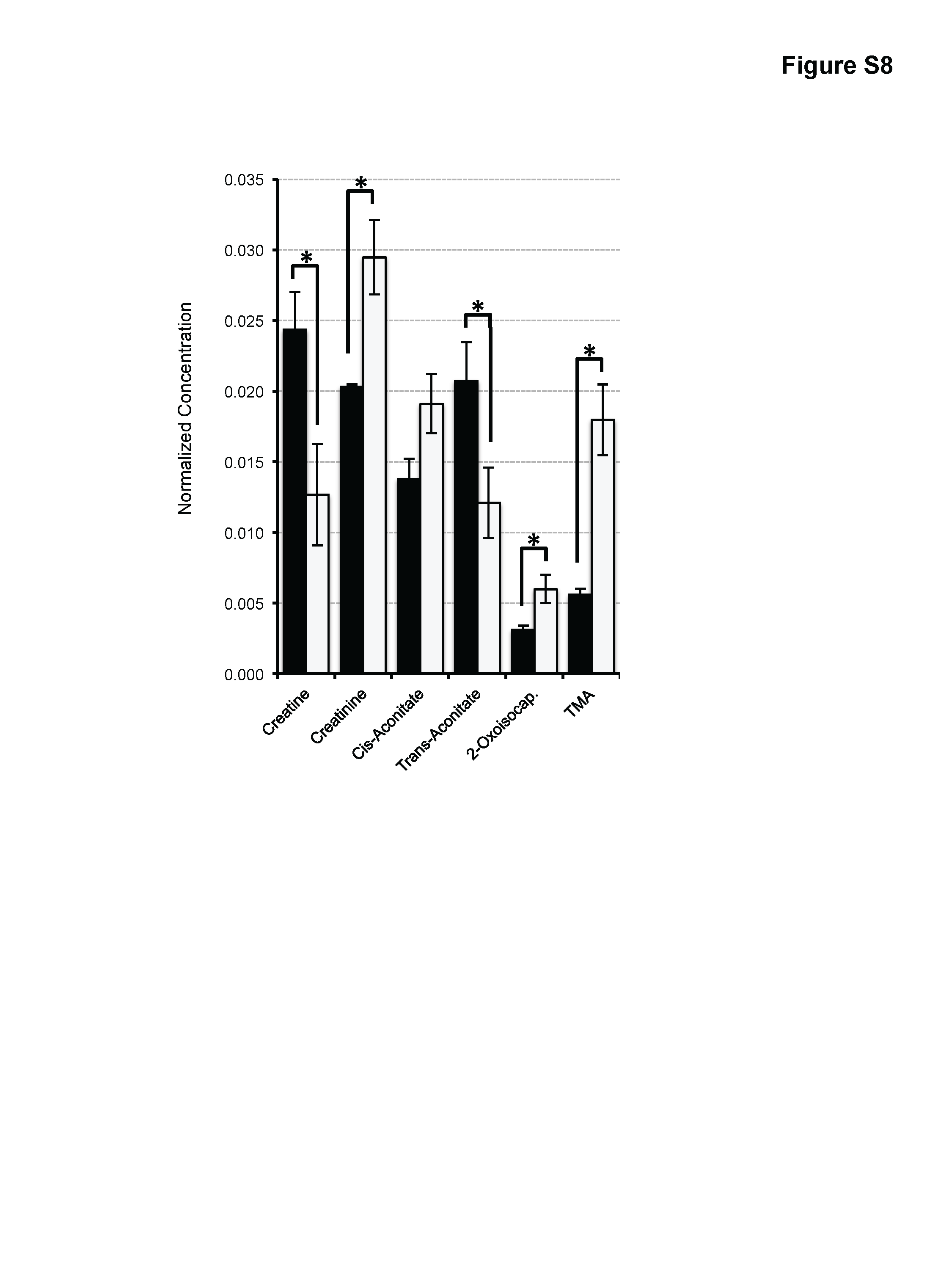

Supplement: S8 Fig — (TIFF) [file pone.0149410.s009.tiff]

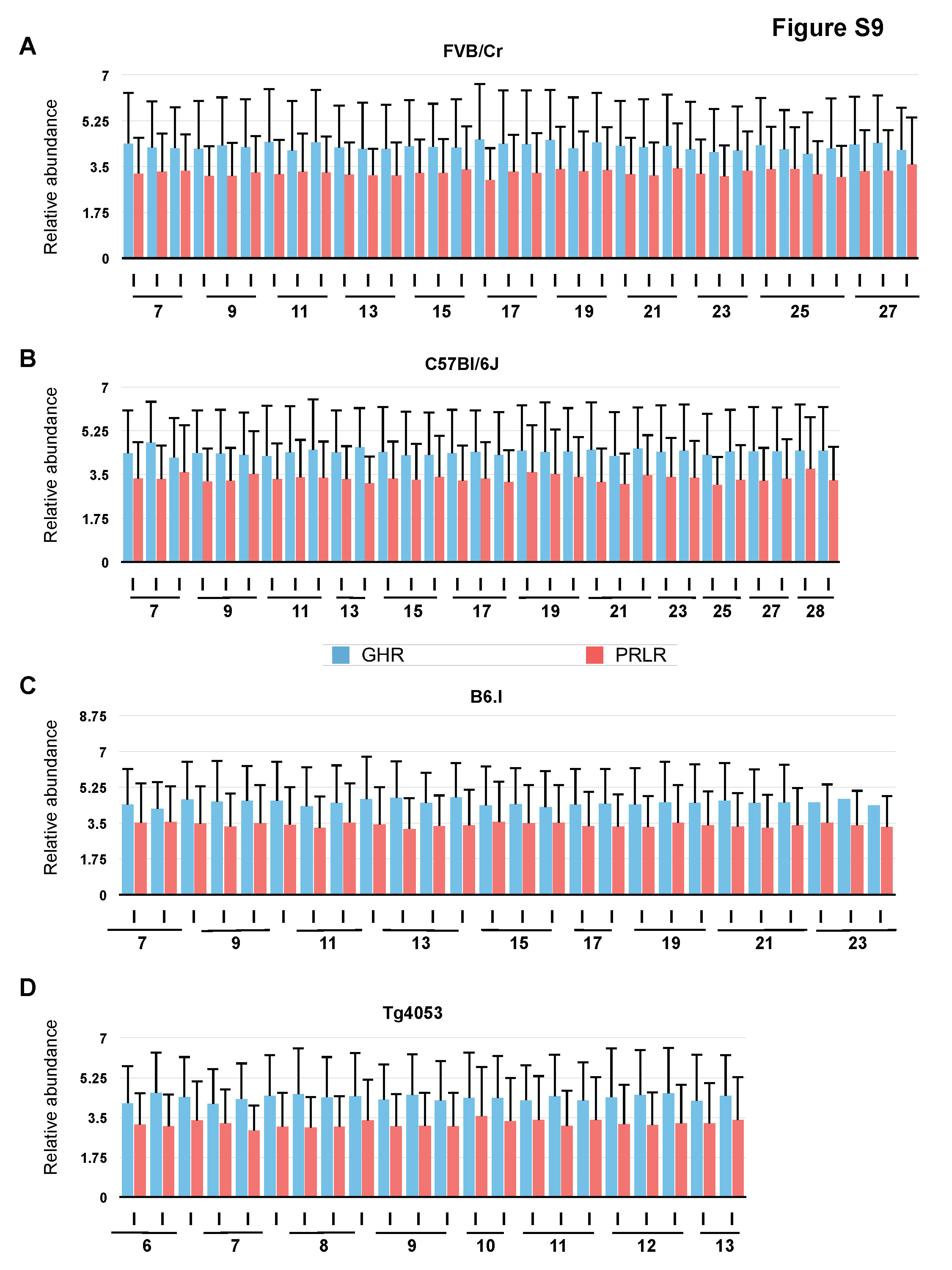

Supplement: S9 Fig — (TIFF) [file pone.0149410.s010.tiff]
