# Supplementary material for: A Common Phenotype Polymorphism in Mammalian Brains Defined by Concomitant Production of Prolactin and Growth Hormone
Source: PLoS One. 2016 Feb 19;11(2):e0149410. doi: 10.1371/journal.pone.0149410 (PMC4760942; doi:10.1371/journal.pone.0149410)
Supplement: S1 Table — (PDF) [file pone.0149410.s011.pdf]

Table S1: Primer sequences used in qRT-PCR

Name	sense	sequence
GAPDH	Forward	3'-TGCCCCCATGTTTGTGATG-5'
	Reverse	3'-TGTGGTCATGAGCCCTTCC-5'
PRL	Forward	3'-GGGTCAGCCCAGAAAGCAG-5'
	Reverse	3'-CAGTCACCAGCGGAACAGATT-5'
GH	Forward	3'-AAGAGTTCGAGCGTGCCTAC-5'
	Reverse	3'-GGATGGTCTCTGAGAAGCAGA-5'
