# Supplementary material for: A Common Phenotype Polymorphism in Mammalian Brains Defined by Concomitant Production of Prolactin and Growth Hormone
Source: PLoS One. 2016 Feb 19;11(2):e0149410. doi: 10.1371/journal.pone.0149410 (PMC4760942; doi:10.1371/journal.pone.0149410)
Supplement: S2 Table — (PDF) [file pone.0149410.s012.pdf]

Table S2: Microarray probe set

Array	Platform	Species	Gh	Prl	RPS29	RPL30	Oaz1
[Mouse430_2] Affymetrix Mouse Genome 430 2.0 Array	GPL1261	Mouse	1437522_x_at	1429287_a_at	1415833_x_at	1418273_a_at	1436292_a_at
			1456595_x_at		1437413_x_at	1438076_at	1428868_a_at
			1460310_a_at		1438859_x_at	1456266_at	
			1460613_x_at				
[RG_U34A] Affymetrix Rat Genome U34 Array	GPL85	Rat	J00739_at	V01250cds_s_at	rc_AA965264_at	D84480_s_at	D10706cds#3_s_at
				V01244_at		rc_AI233749_at	D10706_s_at
				E03166cds			
[HG-U133A] Affymetrix Human Genome U133A Array	GPL96	Human	203807_x_at	205445_at	201094_at	200062_s_at	215952_s_at
			205840_x_at				200077_s_at
			206885_x_at				
			206886_x_at				
			208341_x_at				
			208342_x_at				
			211151_x_at				
	GPL201	Human	203807_x_at	205445_at	201094_at	200062_s_at	200077_s_at
			205840_x_at				
[HG-U133_Plus_2] Affymetrix Human Genome U133 Plus 2.0 Array	GPL570	Human	203807_x_at	205445_at	201094_at	200062_s_at	
			205840_x_at				
			206885_x_at				
			206886_x_at				
			208341_x_at				
			208342_x_at				
			211151_x_at				
[HG-U133A_2] Affymetrix Human Genome U133A 2.0 Array	GPL571	Human	203807_x_at	205445_at	201094_at	200062_s_at	D10706cds#2_s_at
			205840_x_at				
			206885_x_at				
			206886_x_at				
			208341_x_at				
			208342_x_at				
			211151_x_at				
