# Supplementary material for: A Common Phenotype Polymorphism in Mammalian Brains Defined by Concomitant Production of Prolactin and Growth Hormone
Source: PLoS One. 2016 Feb 19;11(2):e0149410. doi: 10.1371/journal.pone.0149410 (PMC4760942; doi:10.1371/journal.pone.0149410)
Supplement: S3 Table — (PDF) [file pone.0149410.s013.pdf]

Table S3: 134 genes associtaed to GH and PRL

MatlabID	ProbesetID	Entrez gene id	Gene Symbol	Description
254	1415923_at	17984	Ndn	necdin
262	1415931_at	16002	Igf2	insulin-like growth factor 2
486	1416155_at	15354	Hmgb3	high mobility group box 3
515	1416184_s_at	15361	Hmga1	high mobility group AT-hook 1
534	1416203_at	11826	Aqp1	aquaporin 1
684	1416353_at	22260	Nr1h2	nuclear receptor subfamily 1, group H, member 2
836	1416505_at	15370	Nr4a1	nuclear receptor subfamily 4, group A, member 1
907	1416576_at	12702	Socs3	suppressor of cytokine signaling 3
1312	1416981_at	56458	Foxo1	forkhead box O1
1313	1416982_at	56458	Foxo1	forkhead box O1
1314	1416983_s_at	56458	Foxo1	forkhead box O1
1545	1417214_at	80718	Rab27b	RAB27b, member RAS oncogene family
1546	1417215_at	80718	Rab27b	RAB27b, member RAS oncogene family
1750	1417419_at	12443	Ccnd1	cyclin D1
1751	1417420_at	12443	Ccnd1	cyclin D1
2181	1417850_at	19645	Rb1	retinoblastoma 1
2209	1417878_at	13555	E2f1	E2F transcription factor 1
2263	1417932_at	16173	Il18	interleukin 18
2285	1417954_at	20604	Sst	somatostatin
2433	1418102_at	15205	Hes1	hairy and enhancer of split 1 (Drosophila)
2646	1418315_at	26423	Nr5a1	nuclear receptor subfamily 5, group A, member 1
2653	1418322_at	12916	Crem	cAMP responsive element modulator
2880	1418549_at	12640	Cga	glycoprotein hormones, alpha subunit
2925	1418594_a_at	17977	Ncoa1	nuclear receptor coactivator 1
2934	1418603_at	54140	Avpr1a	arginine vasopressin receptor 1A
2935	1418604_at	54140	Avpr1a	arginine vasopressin receptor 1A
2964	1418633_at	18128	Notch1	Notch gene homolog 1 (Drosophila)
2965	1418634_at	18128	Notch1	Notch gene homolog 1 (Drosophila)
2971	1418640_at	93759	Sirt1	sirtuin 1 (silent mating type information regulation 2, homolog) 1 (S. cerevisiae)
3087	1418756_at	22044	Trh	thyrotropin releasing hormone
3141	1418810_at	12921	Crhr1	corticotropin releasing hormone receptor 1
3281	1418950_at	13489	Drd2	dopamine receptor 2
3313	1418982_at	12606	Cebpa	CCAAT
3578	1419247_at	19735	Rgs2	regulator of G-protein signaling 2
3579	1419248_at	19735	Rgs2	regulator of G-protein signaling 2
3756	1419425_at	12801	Cnr1	cannabinoid receptor 1 (brain)
3828	1419497_at	12576	Cdkn1b	cyclin-dependent kinase inhibitor 1B
3845	1419514_at	18740	Pitx1	paired-like homeodomain transcription factor 1

3850	1419519_at	16000	Igf1	insulin-like growth factor 1
3899	1419568_at	26413	Mapk1	mitogen-activated protein kinase 1
3951	1419620_at	30939	Pttg1	pituitary tumor-transforming 1
3965	1419634_a_at	14601	Ghrh	growth hormone releasing hormone
3993	1419662_at	18295	Ogn	osteoglycin
3994	1419663_at	18295	Ogn	osteoglycin
4089	1419758_at	18671	Abcb1a	ATP-binding cassette, sub-family B (MDR
4090	1419759_at	18671	Abcb1a	ATP-binding cassette, sub-family B (MDR
4497	1420178_at	20850	Stat5a	Transcribed locus
4716	1420410_at	26424	Nr5a2	nuclear receptor subfamily 5, group A, member 2
4822	1420516_at	50518	a	nonagouti
4862	1420556_at	18429	Oxt	oxytocin
4959	1420653_at	21803	Tgfb1	transforming growth factor, beta 1
5021	1420715_a_at	19016	Pparg	peroxisome proliferator activated receptor gamma
5071	1420765_a_at	20371	Foxp3	forkhead box P3
5215	1420909_at	22339	Vegfa	vascular endothelial growth factor A
5501	1421195_at	12425	Cckar	cholecystokinin A receptor
5550	1421244_at	13982	Esr1	estrogen receptor 1 (alpha)
21828	1437522_x_at	14599	Gh	growth hormone
5688	1421382_at	19116	Prlr	prolactin receptor
5697	1421391_at	22355	Vipr2	vasoactive intestinal peptide receptor 2
5702	1421396_at	18548	Pcsk1	proprotein convertase subtilisin
5750	1421444_at	18667	Pgr	progesterone receptor
5775	1421469_a_at	20850	Stat5a	signal transducer and activator of transcription 5A
5821	1421515_at	14536	Nr6a1	nuclear receptor subfamily 6, group A, member 1
5822	1421516_at	14536	Nr6a1	nuclear receptor subfamily 6, group A, member 1
5888	1421582_a_at	12912	Creb1	cAMP responsive element binding protein 1
5889	1421583_at	12912	Creb1	cAMP responsive element binding protein 1
5971	1421665_a_at	14715	Gnrhr	gonadotropin releasing hormone receptor
6036	1421730_at	26423	Nr5a1	nuclear receptor subfamily 5, group A, member 1
6046	1421740_at	14683	Gnas	GNAS (guanine nucleotide binding protein, alpha stimulating) complex locus
6145	1421839_at	11303	Abca1	ATP-binding cassette, sub-family A (ABC1), member 1
6146	1421840_at	11303	Abca1	ATP-binding cassette, sub-family A (ABC1), member 1
6172	1421866_at	14815	Nr3c1	nuclear receptor subfamily 3, group C, member 1
6173	1421867_at	14815	Nr3c1	nuclear receptor subfamily 3, group C, member 1
6305	1421999_at	22095	Tshr	thyroid stimulating hormone receptor
6440	1422134_at	14282	Fosb	FBJ osteosarcoma oncogene B
6450	1422144_at	16326	Inhbe	inhibin beta E
6508	1422202_at	21834	Thrb	thyroid hormone receptor beta
6510	1422204_at	26361	Avpr1b	arginine vasopressin receptor 1B
6619	1422313_a_at	16011	Igfbp5	insulin-like growth factor binding protein 5
6630	1422324_a_at	19227	Pthlh	parathyroid hormone-like peptide

6843	1422537_a_at	15902	Id2	inhibitor of DNA binding 2
6888	1422582_at	16846	Lep	leptin
6937	1422631_at	11622	Ahr	aryl-hydrocarbon receptor
6957	1422651_at	11450	Adipoq	adiponectin, C1Q and collagen domain containing
7026	1422720_at	16392	Isl1	ISL1 transcription factor, LIM
7034	1422728_at	16322	Inha	inhibin alpha
7085	1422779_at	58994	Smpd3	sphingomyelin phosphodiesterase 3, neutral
7140	1422834_at	16508	Kcnd2	potassium voltage-gated channel, Shal-related family, member 2
7141	1422835_at	16508	Kcnd2	potassium voltage-gated channel, Shal-related family, member 2
7157	1422851_at	15364	Hmga2	high mobility group AT-hook 2
7218	1422912_at	12159	Bmp4	bone morphogenetic protein 4
7255	1422949_at	18125	Nos1	nitric oxide synthase 1, neuronal
7288	1422982_at	11835	Ar	androgen receptor
7310	1423004_at	22354	Vipr1	vasoactive intestinal peptide receptor 1
7392	1423086_at	18145	Npc1	Niemann Pick type C1
7434	1423128_at	11632	Aip	aryl-hydrocarbon receptor-interacting protein
7706	1423400_at	16591	Kl	klotho
7708	1423402_at	12912	Creb1	cAMP responsive element binding protein 1
7733	1423427_at	11516	Adcyap1	adenylate cyclase activating polypeptide 1
8411	1424105_a_at	30939	Pttg1	pituitary tumor-transforming 1
9238	1424932_at	13649	Egfr	epidermal growth factor receptor
9413	1425107_a_at	16880	Lifr	leukemia inhibitory factor receptor
9535	1425229_a_at	21416	Tcf7l2	transcription factor 7-like 2, T-cell specific, HMG-box
9797	1425491_at	12166	Bmpr1a	bone morphogenetic protein receptor, type 1A
9798	1425492_at	12166	Bmpr1a	bone morphogenetic protein receptor, type 1A
9799	1425493_at	12166	Bmpr1a	bone morphogenetic protein receptor, type 1A
9800	1425494_s_at	12166	Bmpr1a	bone morphogenetic protein receptor, type 1A
9856	1425550_a_at	19084	Prkar1a	protein kinase, cAMP dependent regulatory, type I, alpha
10159	1425853_s_at	19116	Prlr	prolactin receptor
10232	1425926_a_at	18424	Otx2	orthodenticle homolog 2 (Drosophila)
10409	1426103_a_at	13983	Esr2	estrogen receptor 2 (beta)
10891	1426585_s_at	26413	Mapk1	mitogen-activated protein kinase 1
10945	1426639_a_at	21416	Tcf7l2	transcription factor 7-like 2, T-cell specific, HMG-box
11366	1427060_at	26417	Mapk3	mitogen-activated protein kinase 3
11661	1427355_at	12310	Calca	calcitonin
11833	1427527_a_at	19227	Pthlh	parathyroid hormone-like peptide
12000	1427694_at	14715	Gnrhr	gonadotropin releasing hormone receptor
12011	1427705_a_at	18033	Nfkb1	nuclear factor of kappa light polypeptide gene enhancer in B-cells 1, p105
12037	1427731_at	14715	Gnrhr	gonadotropin releasing hormone receptor
12088	1427782_a_at	12921	Crhr1	corticotropin releasing hormone receptor 1
12095	1427789_s_at	14683	Gnas	GNAS (guanine nucleotide binding protein, alpha stimulating) complex locus

12307	1428001_at	22095	Tshr	thyroid stimulating hormone receptor
12480	1428174_x_at	16549	Khsrp	KH-type splicing regulatory protein
12611	1428305_at	18549	Pcsk2	proprotein convertase subtilisin
12970	1428664_at	22353	Vip	vasoactive intestinal polypeptide
13131	1428825_at	14536	Nr6a1	nuclear receptor subfamily 6, group A, member 1
13132	1428826_at	14536	Nr6a1	nuclear receptor subfamily 6, group A, member 1
13159	1428853_at	19206	Ptch1	patched homolog 1
13328	1429022_at	11517	Adcyap1r1	adenylate cyclase activating polypeptide 1 receptor 1
40890	1456595_x_at	14599	Gh	growth hormone
13733	1429427_s_at	21416	Tcf7l2	transcription factor 7-like 2, T-cell specific, HMG-box
13734	1429428_at	21416	Tcf7l2	transcription factor 7-like 2, T-cell specific, HMG-box
14714	1430408_at	12286	Cacna1a	calcium channel, voltage-dependent, P
14904	1430598_at	12916	Crem	cAMP responsive element modulator
15153	1430847_a_at	12916	Crem	cAMP responsive element modulator
15684	1431378_at	14815	Nr3c1	nuclear receptor subfamily 3, group C, member 1
16181	1431875_a_at	13555	E2f1	E2F transcription factor 1
16953	1432647_at	13649	Egfr	epidermal growth factor receptor
18351	1434045_at	12576	Cdkn1b	cyclin-dependent kinase inhibitor 1B
18478	1434172_at	12801	Cnr1	cannabinoid receptor 1 (brain)
18821	1434515_at	17977	Ncoa1	nuclear receptor coactivator 1
19482	1435176_a_at	15902	Id2	inhibitor of DNA binding 2
19688	1435382_at	17984	Ndn	necdin
19689	1435383_x_at	17984	Ndn	necdin
19969	1435663_at	13982	Esr1	estrogen receptor 1 (alpha)
20194	1435888_at	13649	Egfr	epidermal growth factor receptor
20297	1435991_at	110784	Nr3c2	nuclear receptor subfamily 3, group C, member 2
20433	1436127_at	12919	Crhbp	corticotropin releasing hormone binding protein
44602	1460310_a_at	14599	Gh	growth hormone
20803	1436497_at	16000	Igf1	Transcribed locus
21119	1436813_x_at	16549	Khsrp	KH-type splicing regulatory protein
21194	1436888_at	18072	Nhlh2	nescient helix loop helix 2
21370	1437064_at	11835	Ar	androgen receptor
21688	1437382_at	11480	Acvr2a	activin receptor IIA
21707	1437401_at	16000	Igf1	insulin-like growth factor 1
21785	1437479_x_at	21386	Tbx3	T-box 3
44905	1460613_x_at	14599	Gh	growth hormone
22057	1437751_at	19017	Ppargc1a	peroxisome proliferative activated receptor, gamma, coactivator 1 alpha
22159	1437853_x_at	17984	Ndn	necdin
22373	1438067_at	18015	Nf1	neurofibromatosis 1
22696	1438390_s_at	30939	Pttg1	pituitary tumor-transforming 1
22789	1438483_at	18125	Nos1	nitric oxide synthase 1, neuronal
22931	1438625_s_at	17984	Ndn	PCTAIRE-motif protein kinase 1

22971	1438665_at	58994	Smpd3	sphingomyelin phosphodiesterase 3, neutral
23245	1438939_x_at	17984	Ndn	necdin
23284	1438978_x_at	17984	Ndn	necdin
23348	1439042_at	11517	Adcyap1r1	adenylate cyclase activating polypeptide 1 receptor 1
23873	1439567_at	21386	Tbx3	T-box 3
23904	1439598_at	56484	Foxo3	forkhead box O3a
23969	1439663_at	19206	Ptch1	Patched homolog 1
24005	1439699_at	18667	Pgr	Transcribed locus
24253	1439947_at	13070	Cyp11a1	cytochrome P450, family 11, subfamily a, polypeptide 1
24612	1440306_at	110784	Nr3c2	Transcribed locus
5605	1421299_a_at	16842	Lef1	lymphoid enhancer binding factor 1
25814	1441508_at	93759	Sirt1	predicted gene, ENSMUSG00000062298
25829	1441523_at	18015	Nf1	neurofibromatosis 1
25941	1441635_at	14536	Nr6a1	nuclear receptor subfamily 6, group A, member 1
25993	1441687_at	22417	Wnt4	wingless-related MMTV integration site 4
26062	1441756_at	21416	Tcf7l2	Transcribed locus
26084	1441778_at	11516	Adcyap1	Adenylate cyclase activating polypeptide 1
26643	1442337_at	11517	Adcyap1r1	adenylate cyclase activating polypeptide 1 receptor 1
26839	1442533_at	110784	Nr3c2	nuclear receptor subfamily 3, group C, member 2
27182	1442876_at	26413	Mapk1	Transcribed locus
27255	1442949_at	18033	Nfkb1	nuclear factor of kappa light chain gene enhancer in B-cells 1, p105
27313	1443007_at	14683	Gnas	Transcribed locus
27406	1443100_at	21834	Thrb	Transcribed locus
27556	1443250_at	19735	Rgs2	regulator of G-protein signaling 2
27681	1443375_at	14683	Gnas	GNAS (guanine nucleotide binding protein, alpha stimulating) complex locus
27703	1443397_at	17977	Ncoa1	nuclear receptor coactivator 1
27744	1443438_at	17977	Ncoa1	Transcribed locus
28070	1443764_x_at	80718	Rab27b	RAB27b, member RAS oncogene family
28308	1444002_at	26927	Foxl2	forkhead box L2
28435	1444129_at	16392	Isl1	ISL1 transcription factor, LIM
28453	1444147_at	18549	Pcsk2	proprotein convertase subtilisin
28706	1444400_at	19645	Rb1	retinoblastoma 1
28761	1444455_at	12286	Cacna1a	calcium channel, voltage-dependent, P
28900	1444594_at	21386	Tbx3	T-box 3
28933	1444627_at	19645	Rb1	retinoblastoma 1
29073	1444767_at	14683	Gnas	Transcribed locus
29398	1445092_at	110784	Nr3c2	nuclear receptor subfamily 3, group C, member 2
29609	1445303_at	12159	Bmp4	bone morphogenetic protein 4
29666	1445360_at	21803	Tgfb1	Transforming growth factor, beta 1
30063	1445757_at	21386	Tbx3	T-box 3
30065	1445759_at	58994	Smpd3	sphingomyelin phosphodiesterase 3, neutral
30178	1445872_at	14536	Nr6a1	nuclear receptor subfamily 6, group A, member 1

30371	1446065_at	16508	Kcnd2	potassium voltage-gated channel, Shal-related family, member 2
30502	1446196_at	15364	Hmga2	high mobility group AT-hook 2
30646	1446340_at	11835	Ar	androgen receptor
30758	1446452_at	21416	Tcf7l2	transcription factor 7 like 2, T cell specific, HMG box
30833	1446527_at	11608	Agtr1b	angiotensin II receptor, type 1b
31062	1446756_at	208188	Ghsr	growth hormone secretagogue receptor
31377	1447071_at	21416	Tcf7l2	transcription factor 7 like 2, T cell specific, HMG box
31404	1447098_at	16880	Lifr	leukemia inhibitory factor receptor
31941	1447635_at	19084	Prkar1a	protein kinase, cAMP dependent regulatory, type I, alpha
32070	1447764_at	16508	Kcnd2	potassium voltage-gated channel, Shal-related family, member 2
32136	1447830_s_at	19735	Rgs2	regulator of G-protein signaling 2
32146	1447840_x_at	13555	E2f1	E2F transcription factor 1
32297	1447991_at	18549	Pcsk2	proprotein convertase subtilisin
32298	1447992_s_at	18549	Pcsk2	proprotein convertase subtilisin
32334	1448029_at	21386	Tbx3	T-box 3
32452	1448152_at	16002	Igf2	insulin-like growth factor 2
32612	1448312_at	18549	Pcsk2	proprotein convertase subtilisin
32856	1448556_at	19116	Prlr	prolactin receptor
32998	1448698_at	12443	Ccnd1	cyclin D1
33104	1448804_at	13070	Cyp11a1	cytochrome P450, family 11, subfamily a, polypeptide 1
33337	1449037_at	12916	Crem	cAMP responsive element modulator
33461	1449161_at	13615	Edn2	endothelin 2
33482	1449182_at	57264	Retn	resistin
33554	1449254_at	20750	Spp1	secreted phosphoprotein 1
33788	1449488_at	18740	Pitx1	paired-like homeodomain transcription factor 1
34005	1449706_s_at	26424	Nr5a2	Nuclear receptor subfamily 5, group A, member 2
34006	1449707_at	26424	Nr5a2	Nuclear receptor subfamily 5, group A, member 2
34121	1449826_a_at	14173	Fgf2	fibroblast growth factor 2
34146	1449851_at	18626	Per1	period homolog 1 (Drosophila)
34176	1449881_a_at	12374	Casr	calcium-sensing receptor
34177	1449882_a_at	12374	Casr	calcium-sensing receptor
34234	1449939_s_at	13386	Dlk1	delta-like 1 homolog (Drosophila)
34481	1450186_s_at	14683	Gnas	GNAS (guanine nucleotide binding protein, alpha stimulating) complex locus
34502	1450207_at	16880	Lifr	leukemia inhibitory factor receptor
34520	1450225_at	16337	Insr	insulin receptor
34521	1450226_at	19116	Prlr	prolactin receptor
34554	1450259_a_at	20850	Stat5a	signal transducer and activator of transcription 5A
34557	1450262_at	56708	Clcf1	cardiotrophin-like cytokine factor 1
34592	1450297_at	16193	Il6	interleukin 6
34656	1450361_at	19127	Prop1	paired like homeodomain factor 1
34687	1450392_at	11303	Abca1	ATP-binding cassette, sub-family A (ABC1), member 1

34739	1450444_a_at	22259	Nr1h3	nuclear receptor subfamily 1, group H, member 3
34766	1450471_at	17127	Smad3	MAD homolog 3 (Drosophila)
34767	1450472_s_at	17127	Smad3	MAD homolog 3 (Drosophila)
34805	1450510_a_at	12286	Cacna1a	calcium channel, voltage-dependent, P
34862	1450567_a_at	12824	Col2a1	collagen, type II, alpha 1
34868	1450573_at	11705	Amh	anti-Mullerian hormone
34902	1450607_s_at	171530	Ucn2	urocortin 2
34990	1450695_at	11622	Ahr	aryl-hydrocarbon receptor
35018	1450723_at	16392	Isl1	ISL1 transcription factor, LIM
35043	1450748_at	58994	Smpd3	sphingomyelin phosphodiesterase 3, neutral
35068	1450773_at	16508	Kcnd2	potassium voltage-gated channel, Shal-related family, member 2
35075	1450780_s_at	15364	Hmga2	high mobility group AT-hook 2
35076	1450781_at	15364	Hmga2	high mobility group AT-hook 2
35077	1450782_at	22417	Wnt4	wingless-related MMTV integration site 4
35090	1450795_at	16866	Lhb	luteinizing hormone beta
35094	1450799_at	11517	Adcyap1r1	adenylate cyclase activating polypeptide 1 receptor 1
35119	1450824_at	19206	Ptch1	patched homolog 1
35291	1450996_at	14308	Fshb	follicle stimulating hormone beta
35299	1451004_at	11480	Acvr2a	activin receptor IIA
35605	1451310_a_at	13039	Ctsl	cathepsin L
35825	1451530_at	13649	Egfr	epidermal growth factor receptor
36024	1451729_at	12166	Bmpr1a	bone morphogenetic protein receptor, type 1A
36139	1451844_at	19116	Prlr	prolactin receptor
36145	1451850_at	19116	Prlr	prolactin receptor
36187	1451892_at	16591	Kl	klotho
36254	1451959_a_at	22339	Vegfa	vascular endothelial growth factor A
36299	1452004_at	12310	Calca	calcitonin
36309	1452014_a_at	16000	Igf1	insulin-like growth factor 1
36327	1452032_at	19084	Prkar1a	protein kinase, cAMP dependent regulatory, type I, alpha
36409	1452114_s_at	16011	Igfbp5	insulin-like growth factor binding protein 5
36820	1452525_a_at	18015	Nf1	neurofibromatosis 1
36824	1452529_a_at	12912	Creb1	cAMP responsive element binding protein 1
36895	1452600_at	225895	Taf6l	TAF6-like RNA polymerase II, p300
36985	1452690_at	16549	Khsrp	KH-type splicing regulatory protein
37399	1453104_at	26413	Mapk1	mitogen-activated protein kinase 1
37708	1453413_at	14683	Gnas	GNAS (guanine nucleotide binding protein, alpha stimulating) complex locus
37891	1453596_at	15902	Id2	inhibitor of DNA binding 2
38155	1453860_s_at	14815	Nr3c1	nuclear receptor subfamily 3, group C, member 1
38608	1454313_at	13649	Egfr	epidermal growth factor receptor
20704	1436398_at	16842	Lef1	Transcribed locus
39255	1454960_at	17127	Smad3	MAD homolog 3 (Drosophila)

39569	1455274_at	50518	a	Transcribed locus, weakly similar to XP_001373728.1 PREDICTED: similar to envelope [Monodelphis domestica]
39942	1455647_at	11835	Ar	androgen receptor
40067	1455772_at	18667	Pgr	progesterone receptor
40087	1455792_x_at	17984	Ndn	necdin
40194	1455899_x_at	12702	Socs3	suppressor of cytokine signaling 3
40507	1456212_x_at	12702	Socs3	suppressor of cytokine signaling 3
40690	1456395_at	19017	Ppargc1a	peroxisome proliferative activated receptor, gamma, coactivator 1 alpha
40816	1456521_at	26424	Nr5a2	Transcribed locus
40870	1456575_at	17984	Ndn	Necdin
25066	1440760_at	16842	Lef1	lymphoid enhancer binding factor 1
41858	1457563_at	13649	Egfr	epidermal growth factor receptor
41930	1457635_s_at	14815	Nr3c1	nuclear receptor subfamily 3, group C, member 1
42019	1457724_at	13039	Ctsl	cathepsin L
42172	1457877_at	13982	Esr1	Transcribed locus
42279	1457984_at	12918	Crh	corticotropin releasing hormone
42462	1458167_at	16508	Kcnd2	potassium voltage-gated channel, Shal-related family, member 2
42489	1458194_at	13070	Cyp11a1	cytochrome P450, family 11, subfamily a, polypeptide 1
42574	1458279_at	56484	Foxo3	Transcribed locus
42607	1458312_at	12286	Cacna1a	Transcribed locus
42833	1458538_at	93759	Sirt1	Transcribed locus
42921	1458626_at	18125	Nos1	Nitric oxide synthase 1, neuronal
43083	1458788_at	18015	Nf1	Transcribed locus
43465	1459170_at	56458	Foxo1	forkhead box O1
43583	1459288_at	16508	Kcnd2	potassium voltage-gated channel, Shal-related family, member 2
44289	1459996_at	12286	Cacna1a	Calcium channel, voltage-dependent, P
44348	1460055_at	18015	Nf1	Transcribed locus
44595	1460303_at	14815	Nr3c1	nuclear receptor subfamily 3, group C, member 1
39029	1454734_at	16842	Lef1	lymphoid enhancer binding factor 1
44628	1460336_at	19017	Ppargc1a	peroxisome proliferative activated receptor, gamma, coactivator 1 alpha
44712	1460420_a_at	13649	Egfr	epidermal growth factor receptor
44874	1460582_x_at	50518	a	Transcribed locus, weakly similar to XP_001373728.1 PREDICTED: similar to envelope [Monodelphis domestica]
44883	1460591_at	13982	Esr1	estrogen receptor 1 (alpha)
13593	1429287_a_at	19109	Prl	prolactin
