# Supplementary material for: A Common Phenotype Polymorphism in Mammalian Brains Defined by Concomitant Production of Prolactin and Growth Hormone
Source: PLoS One. 2016 Feb 19;11(2):e0149410. doi: 10.1371/journal.pone.0149410 (PMC4760942; doi:10.1371/journal.pone.0149410)
Supplement: S4 Table — (PDF) [file pone.0149410.s014.pdf]

Table S4 Genes location

MATLABID	Probe Set ID	Symbol	Gene Description	Gene Accession	Cytoband	UniGene ID	Chr	Strand	Start	Stop	SwissProt mRna Access	SwissProt access
4765	10392135	Gh	growth hormone	NM_008117	11 D|11 65.0 cM	Mm.343934	chr11	-	106161582	106163124	NM_008117	P06880
6056	10404264	Prl	prolactin	NM_011164	13 A3.1|13 14.0 cM	Mm.1270	chr13	+	27149445	27157061	NM_011164	P06879
16609	10503947	Cga	glycoprotein hormones, alpha subunit	NM_009889	4 A5|4 10.5 cM	Mm.1361	chr4	+	34841028	34854623	BC087926	P01216
4937	10394240	Pomc	pro-opiomelanocortin-alpha	NM_008895	12 A1.1|12 4.0 cM	Mm.277996	chr12	+	3954951	3960618	NM_008895	P01193
