# Supplementary material for: A Common Phenotype Polymorphism in Mammalian Brains Defined by Concomitant Production of Prolactin and Growth Hormone
Source: PLoS One. 2016 Feb 19;11(2):e0149410. doi: 10.1371/journal.pone.0149410 (PMC4760942; doi:10.1371/journal.pone.0149410)
Supplement: S6 Table — (PDF) [file pone.0149410.s016.pdf]

Table S6: miRNAs associated with expression of GH and PRL transcripts

miRNA	gene	source
miR-203	PRL	Integrated Database
miR-223	PRL	Integrated Database, IPA* (-3p)
miR-143	PRL	Integrated Database
miR-299-3p	PRL	Integrated Database
miR-381	PRL	Integrated Database
miR-300	PRL	Integrated Database
miR-4328	PRL	Integrated Database, IPA
miR-490-5p	PRL	Integrated Database
miR-5702	PRL	Integrated Database
miR-590-3p	GH	Integrated Database
miR-138	GH	Integrated Database
miR-541-3p	GH	IPA
miR-3594-5p	GH	IPA
miR-4640-5p	GH	IPA
miR-891b	GH	IPA
miR-3918	GH	IPA
miR-4327	PRL	IPA
miR-769-5p	PRL	IPA
miR-3150-3p	PRL	IPA
miR-4521	PRL	IPA

*IPA: Ingenuity® pathway analysis
