# Supplementary material for: A Common Phenotype Polymorphism in Mammalian Brains Defined by Concomitant Production of Prolactin and Growth Hormone
Source: PLoS One. 2016 Feb 19;11(2):e0149410. doi: 10.1371/journal.pone.0149410 (PMC4760942; doi:10.1371/journal.pone.0149410)
Supplement: S1 Appendix — (DOCX) [file pone.0149410.s017.docx]

**Supplementary Material**

**Supplementary methods**

**Analysis of GH and PRL content in serum samples**

A total of 38 FVB/NCr mice consisting of 12 females aged 131 to 176 days and 26 males aged 131 to 161 days were used. Serum GH and PRL were measured using a commercial multiplex immunoassay kit (MPTMAG-49K, Millipore-EMD Billerica, MA, USA) at The Analytical Facility for Bioactive Molecules, Toronto, ON, Canada. Samples were diluted 2.5x and run according to manufacturer’s instructions. Samples were analysed by Luminex 200 (Luminex Corp., Austin, TX) and xPonent 3.1.971.0 software. Each sample was done in duplicate.

**Sample collection and preparation for tube-in-tube ^1^H-NMR spectroscopy**

Urine was collected from individual mice, flash frozen in dry ice and stored at -80˚C. All urine samples were blinded for downstream ^1^H-NMR spectroscopy data collection and processing. Samples were prepared for tube-in-tube ^1^H-NMR spectroscopy as previously described [[1](#_ENREF_1)]. Briefly, 10 μL of buffer (120 mM sodium phosphate, pH 7.5 with 0.3% sodium azide) was added per 50 μL of mouse urine. Buffered urine samples were spun at 4000 x*g* for 5 min to clear insoluble materials. Clarified urine samples were filtered using Nanosep 3K Omega centrifugal filters (3-kDa molecular weight cut-off; Pall). Filters were twice prewashed with 400 μL of 0.05 N NaOH, twice prewashed with 400 μL distilled water, followed by removal of wash retentate. A small volume of the sample filtrate (~30 μL) was loaded into a glass melting point capillary (0.8 - 1.1 mm x 90 mm; Kimble Chase) using a custom Hamilton syringe (Hamilton Company). The reference solution for NMR was 0.5 mM deuterated 2,2-dimethyl-2-silapentane-5-sulfonic acid (DSS-d_6_; Chenomx Inc., Edmonton, AB, Canada) in 99.9% D2O. Sample loaded capillaries were placed in 5 mm NMR tubes containing ~530 μL of reference solution and subjected to a low-speed spin to remove sample air bubbles. Care was taken to ensure the sample height inside the capillary matched the final height of the reference solution outside after the sample-loaded capillary was inserted (+/- 2 mm).

**^1^H-NMR spectroscopy and data processing**

All one-dimensional ^1^H-NMR spectra were acquired at 303 K on a 500 MHz (AR) premium-shielded Agilent spectrometer using the tnnoesy pulse sequence with water pre-saturation in VnmrJ v3.2 (100 ms mixing time; 1 s recycling delay; 4 s acquisition; sweep width 12.0 ppm; 256 transients). The 90° pulse-width was chosen to maximize the intensity of the DSS peak. A shim-map made using a tube-in-tube sample was used for gradient shimming. NMR spectra were manually phased using VnmrJ v3.2 and imported into Chenomx NMR Suite v7.0. Background subtraction was performed using the cubic-spline-based baseline adjustment. Spectra were referenced to the 2-oxoisocaproate doublet at ~0.90 ppm, because the chemical shift of the external DSS in the tube-in-tube set-up requires correction [[1](#_ENREF_1)]. Data were binned into 217 bins along the chemical shift axis with the water, urea and DSS-d_6_ regions excluded (average 0.02 ppm wide). Resonance peaks with variable chemical shifts among overlapped spectra were custom binned into larger data bins (to a maximum size of 0.08 ppm) such that there were no overlapping bins. Data bins were normalized to the total binned signal to account for differences in urine concentration.

**Multivariate statistics and metabolite quantification**

Data bins were imported into SIMCA P+ v12.0.1 (Umetrics) for multivariate statistical analyses. Following pareto-scaling to include bins with high and low signals while minimizing the effect of noise [[2](#_ENREF_2), [3](#_ENREF_3)], the entire data set was assessed by Principal Component Analysis (PCA) and outliers removed (data points outside of Hotelling’s T2 ellipse using the first 2 components). Data were then analyzed for discrimination between high and low GH/PRL classes using orthogonal projection to latent structures discriminant analysis (OPLS-DA). Urine samples were modeled in a gender-dependent manner to allow interpretation of variables correlated to inter-class variation. Based on the preliminary data, male mouse urine data generated OPLS-DA models with good model indicator values of inter-class variation (R2Y), goodness of prediction (Q2), and predictive ability based on internal hold-out samples. Female mouse urine data did not generate good OPLS-DA model indicators, likely because of limited, suitable sample numbers. Data bins responsible for class discrimination of male GH-positive and GH-negative urine samples were identified using Variable Importance for Projection scores and the S-plot. Identified discriminating data bins were selected for target profiling and quantification of the constituent urinary metabolites in the background-subtracted NMR spectra using Chenomx NMR Suite v7.0. The integral of the DSS-d_6_ methyl proton peak was set to 0.5 mM (the final DSS concentration in the reference solution). Profiled metabolite concentrations were normalized to the total binned signal to account for inter-sample variation in urine concentration. Statistical analyses of normalized metabolite concentrations were performed with the two-tailed Student’s t test (unequal variance) using Microsoft Excel 2010. Differences were considered significant when p < 0.05.

**Metabolite profiling of concordant GH/PRL expressers**

Based upon the known trophic effects of GH/PRL and the recent understanding of bidirectional signaling between the brain and gut microbiome [[4](#_ENREF_4)], we considered whether the subset of animals that overproduce GH and PRL in the CNS might be distinguished by profiling metabolites in biofluids. To this end we examined urine, which contains signatures of endogenous and microbiome metabolites. Urine collected from mice at the time of sacrifice was used in a tube-in-tube method for NMR determination of metabolite content in microliter volumes of unprocessed samples (**S8 Fig)**, with brains then removed, homogenized and phenotyped for protein expression by western blot analysis as above; this analysis of GH and PRL protein levels informed statistical analyses of urine NMR spectra by assigning blinded urine samples into two groups, but did not provide the respective hormone levels of each group. After separating urine samples based on sex, multi-variate statistical analyses of spectral data of urine samples revealed that the groupings of animals were distinguishable and the *post hoc* analysis of GH and PRL indicated that group 1 and group 2 corresponded to animals with baseline or elevated CNS GH + PRL, respectively. Based on OPLS-DA modeling, male mouse urine data generated good indicator values of inter-class variation (cumulative R2Y: 0.80), intra-class variation (cumulative R2X: 0.58), goodness of prediction (Q2: 0.53), and also predictive ability based on internal “hold-out” samples (4 out of 5 unknown-class spectra and 10 out of 10 known-class spectra were correctly modeled) using two-thirds of the data as a training set and one-third as a hold-out set [[5](#_ENREF_5)]. Although reasonable indicators for female mice intra-class (cumulative R2Y: 0.62) and inter-class (cumulative R2X: 0.57) variation were modeled using OPLS-DA, the goodness of prediction (Q2: 0.04) and hold-out sample predictive ability were weak (4 out of 5 unknown-class spectra and 9 out of 10 known-class spectra correctly modeled), perhaps due to a smaller sample size. Next, we sought to determine the metabolites that underpinned the class discrimination achieved for the male mice. Metabolites were profiled by spectral inspection versus a reference library and their absolute concentrations normalized to the total NMR signal to account for differences in urine concentration. Normalized concentrations were divided empirically into metabolites present at high, medium and low abundance (**S8 Fig**). Within these groups, distinctions reached significance (p < 0.05) in male mice in the medium (creatine, creatinine and *trans*-aconitate) and low metabolite concentration groups (2-oxoisocaproate and trimethylamine). These data establish a potential for class discrimination by a non-invasive method and, along with behavioral data, suggest that stable phenotypic states are associated with or caused by the molecular polymorphism in extra-pituitary PRL/GH levels.

**Supplementary Figure Legends**

**S1 Fig. GH and PRL transcripts in spleen analyzed in healthy cohorts of four different mouse strains**

Expression profiles of GH (4 different probe sets) and PRL (1 probe set) in spleen of individual mice of different background during aging is shown. Dynamic gene expression profiles of (A) mice of FVB/Cr background aged from 7 to 27 weeks (n=26), (C) mice of C57Bl/6J background, aged from 7 to 27 weeks (n=29), (E) mice of B6.I *Prnp*^b^ background aged from 7 to 33 weeks (n=21). Covariance (y-axis) versus correlation coefficient plots are shown in the corresponding right-hand panels (B, D, F)

**S2 Fig. Linear regression between GH and PRL expression in rat brain**

Linear regression analyses of PRL (y-axis) and GH (x-axis) transcript levels in brain RNAs from Sprague Dawley (A) Wistar (B) and Wistar Kyoto (C) rat are presented.

**S3 Fig. Linear regression between GH and PRL expression in diverse human brain structures**

Linear regression analyses of PRL (y-axis) and GH (x-axis) transcript levels in brain RNAs from Amygdala (A), cerebellum (B), frontal lobe (C), hippocampus (D), and putamen (E) are presented.

**S4 Fig. Other aspects of water maze performance for high-expressing GH/PRL mice**

(A) During a probe trial administered at the end testing, 10 month-old high-expressing GH/PRL mice (black shading) swam faster than the low-expressing counterparts. (B) High GH/PRL expressing females showed a tendency of faster swim speed than the low expresser females, while the opposite trend was noted in the swim speeds of low and high expresser males.

**S5 Fig. Control western blot for antibody specificity**

Protein expression of GH in mouse brains. Two sets of brain protein samples (50μg total protein) from two low expression and two high expression animals were electrophoresed and immunoblotted. The left hand side of the gel was processed with primary and secondary antibodies while the right hand side of the gel was probed with the secondary antibody alone. The (control) lane represents 4 ng of pituitary extract as positive control.

**S6 Fig. Comparison of GH and PRL content in mouse brain versus serum**

Linear regression analyses of serum (y-axis) and brain (x-axis) hormones levels in 38 FVB/NCr mice for GH (A) and PRL (B).

**S7 Fig. Immunohistochemical analyses of mice with basal or elevated extra-pituitary GH/PRL expression**

Fluorescent double staining for PRL (green, panels 1, 4, 7, 10), GH (red, panels 2, 5, 8, 11) and merged expression (orange, panels 3, 6, 9, 12) is presented. Panels 1-3 and 7-9 correspond to the brain of an animal which is low expresser of GH/PRL by western blot while panels 4-6 and 10-12 correspond to the brain of an animal which is defined as a high expresser of GH/PRL by western blot. Different regions of the brain are shown: cerebellum (1 to 6) and amygdala (7 to 12). Scale bars represent 25 μM.

**S8 Fig. Metabolomic analyses of mice with basal or elevated extra-pituitary GH/PRL expression**

Normalized metabolite concentrations in urine of GH/PRL high expresser (black bars; n = 5) and GH/PRL low expresser (white bars; n = 7) male mice. Data represent profiled metabolite concentrations normalized to the total binned NMR signal (to account for inter-sample variations in urine concentration). Values are represented as mean ± SEM. PCA and OPLS-DA were used to initially identify regions of NMR urine spectra that discriminated GH-positive and GH-negative male urine samples. Constituent urinary metabolites of the discriminating NMR spectral regions were then profiled and quantified using Chenomx NMR Suite v7.0. 2-oxoisocap – 2-oxoisocaproate; TMA – Trimethylamine. Statistical analyses were performed using the two-tailed Student t test with unequal variance. For all panels significant differences (p < 0.05) are denoted by an asterisk.

**S9 Fig. Microarray analysis of brain GH and PRL receptors transcripts in 113 individual mice**

Expression profiles of GH receptor (blue) and PRL (red) in brain of individual mice of different genetic background during aging are shown. Dynamic gene expression profiles of (A) mice of FVB/NCr background aged from 7 to 27 weeks (n=34), (B) mice of C57Bl/6J background, aged from 7 to 28 weeks (n=31), (C) mice of B6.I *Prnp*^b^ background aged from 7 to 23 weeks (n=26), and (D) data from Tg*Prnp*^a^(4053) mice are shown aged from 6 to 13 weeks (n=22).

**S1 Table. Primer sequences used in qRT-PCR**

**S2 Table. Microarray probe-sets**

**S3 Table. 134 genes associated with GH and PRL**

**S4 Table. Data on human controls**

**S5 Table. Gene locations**

**S6 Table. miRNAs associated with expression of GH and PRL transcripts**

**APPENDIX 1: Extended discussion of alternative explanations for concomitant extrapituitary GH and PRL expression**

In parallel to discussion of the implications of coordinate expression presented in the main text it is useful to reprise technical aspects of the studies, to consider all possible confounds or sources of experimental error that might pertain to our investigations.

**Reagent specificity**. The presented mouse microarray studies involve 6 independent probe sets for GH and one for PRL, with distinct oligonucleotide primers used for corroborative RT-PCR studies of mouse transcripts and yet different set of probes employed for studies of human transcripts. Probes for 29 other GH- and PRL-like genes failed to reveal concomitant expression with GH and PRL. Antibodies used for the detection of rodent GH and PRL derive from an NIH resource and are used as the basis for radioimmunoassays [[6](#_ENREF_6)] of these two hormones, which retain only 9% sequence identity in amino acid sequence. GH and PRL immunoreactive species by blot analysis of brain tissue were of the correct molecular weight (26 kDa) as predicted from DNA sequence analysis and as confirmed by co-electrophoresis of recombinant GH and PRL standards (not shown) and distinct by ~10 kDa from a 16 kDa POMC N-terminal fragment reported in one study to cross-react with PRL antibodies [[7](#_ENREF_7)]. We also note that mouse PRL and POMC share only 6.8% sequence identity. Potential artifacts arising from cross-reactivity between secondary antibodies and endogenous rodent immunoglobulins are unlikely as the primary antibodies for blot analyses were rabbit polyclonals, i.e. use cognate anti-rabbit immunoglobulin secondary antibodies for immunodetection and, furthermore, the secondary antibody applied in the absence of primary antibody produced negligible signals (**S5** **Fig**). We also note that immuno-histochemical studies were performed with alternative reagents, monoclonal antibodies, but yet defined neuronal expression (**Fig 6A**). In sum, off-target effects from nucleic acid or protein directed reagents seem unlikely as an explanation for our results; this interpretation is also corroborated by analyses showing concordance between RNA and protein-based measures of gene expression in samples from the same animals (**Fig 4**).

**Cross-contamination**. To consider cross-contamination by blood control experiments used saline perfusion of brains to dilute blood-borne proteins before the preparation of cell lysates still revealed a sub-set of “high expresser” animals (**e.g. Figs 4, 5C**). Cross-contamination from pituitary tissue is important to consider as pituitary and putative extra-pituitary GH and PRL seem to be indistinguishable with regards to their mRNA and protein sequences [[8-10](#_ENREF_8)]. In our procedures the pituitary is routinely left in the brainpan during standard dissections for removal of brains for RNA preparation and, while contamination might occur on an occasional basis for rodent experiments, this effect was specifically addressed in experiments involving the dissection of brain quadrants, analyses that still revealed a subset of high-expresser animals (**Fig 5C, D**). In humans the increased size of the brain (~3500x larger than that of a mouse; 1400 g vs 0.4 g) means that dissected regions are physically dispersed such that cross-contamination effects become a remote possibility. Furthermore, the aforementioned immunostaining within neuronal cell bodies (**Fig 6A**) speaks against hypothetical cross-contamination onto the surface of excised tissue specimens. Lastly, there are prior, albeit (crucially) separate - literatures for extra-pituitary expression of GH and PRL in CNS neurons [[11-14](#_ENREF_11)]. The presence of GH in the hippocampus as documented here (**Fig** **6A**) is consistent with the finding of others [[15](#_ENREF_15)]. Similarly, above and beyond the concept of circulating PRL having action upon CNS progenitor cells [[16](#_ENREF_16)], extra-pituitary PRL expression in CNS neurons has been reported by several laboratories [[11-14](#_ENREF_11)].

**Biological variation.** How might the coordinate overexpression effect arise? While estrus can be entertained in discussions of extra-pituitary PRL fluctuations, documentation of this effect within females beyond their reproductive prime and the presence of PRL high expresser male rodents (**Fig 3**, **Table 3)** and humans (**Fig** **8**) argues against this mechanism. Similarly, while diurnal rhythms for extra-pituitary GH and PRL could be considered, the brain samples for microarray studies were in all cases retrieved during the light cycle. In terms of accepted aspects of the biology of pituitary GH there is a decline of GH/IGF1 axis with age [[17](#_ENREF_17)], which, amongst other effects, induces a decline in neurogenesis; however we do not see a decrease with age in the expression of both hormones in high expresser rodents and a trend to the contrary was present in the human microarray data (**Fig 8**). Extra-pituitary expression of GH or PRL (as individually regulated entities) has been equated with a mechanism to redress deficits in signaling deriving from circulating hormones when pituitary function is attenuated [[7](#_ENREF_7), [12](#_ENREF_12), [13](#_ENREF_13), [18-20](#_ENREF_18)] but since our experiments did not involve chemical or surgical ablation of pituitary function these concepts are inapplicable. For the extra-pituitary expression of GH and PRL defined here in a subset of housed rodents or in humans the more general question arises as to whether we are sampling outliers in a spectrum of physiological variation of gene expression. While we cannot exclude this is the case for the individual hormones, since GH and PRL are non-syntenic (**S5 Table**) and serve different functions, parallel overexpression (with discordances no greater than 12%; **Table 3**) argues for a coordinated biological phenomenon, not coinciding outliers.

**References**

1. Glaves JP, Li MX, Mercier P, Fahlman RP, Sykes BD. High-throughput, multi-platform metabolomics on very small volumes: 1H NMR metabolite identification in an unadulterated tube-in-tube system. Metabolomics. 2014;10:1145-51.

2. van den Berg RA, Hoefsloot HC, Westerhuis JA, Smilde AK, van der Werf MJ. Centering, scaling, and transformations: improving the biological information content of metabolomics data. BMC Genomics. 2006;7:142. doi: 10.1186/1471-2164-7-142. PubMed PMID: 16762068; PubMed Central PMCID: PMC1534033.

3. Worley B, Powers R. Multivariate Analysis in Metabolomics. Current Metabolomics. 2013;1:92-107.

4. Mayer EA, Knight R, Mazmanian SK, Cryan JF, Tillisch K. Gut microbes and the brain: paradigm shift in neuroscience. J Neurosci. 2014;34(46):15490-6. doi: 10.1523/JNEUROSCI.3299-14.2014. PubMed PMID: 25392516; PubMed Central PMCID: PMC4228144.

5. Xia J, Broadhurst DI, Wilson M, Wishart DS. Translational biomarker discovery in clinical metabolomics: an introductory tutorial. Metabolomics. 2013;9(2):280-99. doi: 10.1007/s11306-012-0482-9. PubMed PMID: 23543913; PubMed Central PMCID: PMC3608878.

6. Parlow AF. National Hormone and Peptide Program. Endocrinology. 2002;143(5):1975-7. doi: 10.1210/endo.143.5.0134. PubMed PMID: 11973810.

7. Harlan RE, Shivers BD, Fox SR, Kaplove KA, Schachter BS, Pfaff DW. Distribution and partial characterization of immunoreactive prolactin in the rat brain. Neuroendocrinology. 1989;49(1):7-22. PubMed PMID: 2716951.

8. Render CL, Hull KL, Harvey S. Neural expression of the pituitary GH gene. J Endocrinol. 1995;147(3):413-22. PubMed PMID: 8543911.

9. Wilson DM, 3rd, Emanuele NV, Jurgens JK, Kelley MR. Prolactin message in brain and pituitary of adult male rats is identical: PCR cloning and sequencing of hypothalamic prolactin cDNA from intact and hypophysectomized adult male rats. Endocrinology. 1992;131(5):2488-90. doi: 10.1210/endo.131.5.1339346. PubMed PMID: 1339346.

10. Ben-Jonathan N, Mershon JL, Allen DL, Steinmetz RW. Extrapituitary prolactin: distribution, regulation, functions, and clinical aspects. Endocr Rev. 1996;17(6):639-69. PubMed PMID: 8969972.

11. Fuxe K, Hokfelt T, Eneroth P, Gustafsson JA, Skett P. Prolactin-like immunoreactivity: localization in nerve terminals of rat hypothalamus. Science. 1977;196(4292):899-900. PubMed PMID: 323973.

12. Hojvat S, Emanuele N, Baker G, Connick E, Kirsteins L, Lawrence AM. Growth hormone (GH), thyroid-stimulating hormone (TSH), and luteinizing hormone (LH)-like peptides in the rodent brain: non-parallel ontogenetic development with pituitary counterparts. Brain Res. 1982;256(4):427-34. PubMed PMID: 7127150.

13. DeVito WJ. Distribution of immunoreactive prolactin in the male and female rat brain: effects of hypophysectomy and intraventricular administration of colchicine. Neuroendocrinology. 1988;47(4):284-9. PubMed PMID: 3374755.

14. Johansson JO, Larson G, Andersson M, Elmgren A, Hynsjo L, Lindahl A, et al. Treatment of growth hormone-deficient adults with recombinant human growth hormone increases the concentration of growth hormone in the cerebrospinal fluid and affects neurotransmitters. Neuroendocrinology. 1995;61(1):57-66. PubMed PMID: 7537355.

15. Donahue CP, Kosik KS, Shors TJ. Growth hormone is produced within the hippocampus where it responds to age, sex, and stress. Proc Natl Acad Sci U S A. 2006;103(15):6031-6. Epub 2006/04/01. doi: 0507776103 [pii]

10.1073/pnas.0507776103. PubMed PMID: 16574776; PubMed Central PMCID: PMC1420633.

16. Shingo T, Gregg C, Enwere E, Fujikawa H, Hassam R, Geary C, et al. Pregnancy-stimulated neurogenesis in the adult female forebrain mediated by prolactin. Science. 2003;299(5603):117-20. Epub 2003/01/04. doi: 10.1126/science.1076647

299/5603/117 [pii]. PubMed PMID: 12511652.

17. Pathipati P, Gorba T, Scheepens A, Goffin V, Sun Y, Fraser M. Growth hormone and prolactin regulate human neural stem cell regenerative activity. Neuroscience. 2011;190:409-27. doi: 10.1016/j.neuroscience.2011.05.029. PubMed PMID: 21664953.

18. Toubeau G, Desclin J, Parmentier M, Pasteels JL. Cellular localization of a prolactin-like antigen in the rat brain. J Endocrinol. 1979;83(2):261-6. PubMed PMID: 392042.

19. Thompson SA. Localization of immunoreactive prolactin in ependyma and circumventricular organs of rat brain. Cell Tissue Res. 1982;225(1):79-93. PubMed PMID: 7116429.

20. Paut-Pagano L, Roky R, Valatx JL, Kitahama K, Jouvet M. Anatomical distribution of prolactin-like immunoreactivity in the rat brain. Neuroendocrinology. 1993;58(6):682-95. PubMed PMID: 8127395.
